# An Individual-Based Model of Zebrafish Population Dynamics Accounting for Energy Dynamics

**DOI:** 10.1371/journal.pone.0125841

**Published:** 2015-05-04

**Authors:** Rémy Beaudouin, Benoit Goussen, Benjamin Piccini, Starrlight Augustine, James Devillers, François Brion, Alexandre R. R. Péry

**Affiliations:** 1 Unité Modèles pour l’Ecotoxicologie et la Toxicologie (METO), Institut National de l’Environnement Industriel et des Risques (INERIS), Verneuil en Halatte, France; 2 Unité Ecotoxicologie in vitro et in vivo (ECOT), Institut National de l’Environnement Industriel et des Risques (INERIS), Verneuil en Halatte, France; 3 Centre for Ocean Life, National Institute of Aquatic Resources, Technical University of Denmark, Charlottenlund, Denmark; 4 CTIS, Rillieux La Pape, France; 5 AgroParisTech, Paris, France; Towson University, UNITED STATES

## Abstract

Developing population dynamics models for zebrafish is crucial in order to extrapolate from toxicity data measured at the organism level to biological levels relevant to support and enhance ecological risk assessment. To achieve this, a dynamic energy budget for individual zebrafish (DEB model) was coupled to an individual based model of zebrafish population dynamics (IBM model). Next, we fitted the DEB model to new experimental data on zebrafish growth and reproduction thus improving existing models. We further analysed the DEB-model and DEB-IBM using a sensitivity analysis. Finally, the predictions of the DEB-IBM were compared to existing observations on natural zebrafish populations and the predicted population dynamics are realistic. While our zebrafish DEB-IBM model can still be improved by acquiring new experimental data on the most uncertain processes (e.g. survival or feeding), it can already serve to predict the impact of compounds at the population level.

## Introduction

Data used to estimate the likelihood of adverse ecological effects typically include responses of survival, growth, or reproduction of individuals measured after a specific exposure duration under constant laboratory conditions and in absence of ecological stress (e.g. predation and competition) [1]. These organism-level endpoints are far from the ecological features that the process aims to protect. Indeed, ecological risk assessment should protect the long-term persistence of populations of species in space and time under naturally varying field conditions and in the presence of other stressors (e.g. food limitation). However, except the ecotoxicological data provided by mesocosm experiments and a few field studies [2–5], data on impacts of chemical substances on populations or higher biological levels are very sparse. In this context, population models can play an important role in bridging the gap between what is measured (organism-level endpoints) and what needs to be protected (population-level endpoints) [6].

Zebrafish (*Danio rerio*) offers many practical and methodological advantages that make this small fish species an attractive vertebrate model now used worldwide in a variety of biological disciplines ranging from basic developmental biology to applied toxicology [7]. In the last few years, the popularity of the zebrafish model for investigating chemical ecotoxicity increased notably to address the issues posed by Endocrine Disrupting Chemicals (EDCs) and their reproductive and developmental effects in vertebrate species. This lead to significant knowledge and methodological advances to assess modes of action and effects of EDCs [8–11].

While these experimental approaches can provide relevant information regarding the capacity of chemicals to disrupt key endocrine-regulated physiological processes at organism level, they are limited to address impact at population level. Hence, the development of population dynamics model for zebrafish appears crucial in order to extrapolate from toxicity data measured on organism to biological levels relevant to support and enhance ecological risk assessment [6]. Individual-based models (IBMs) are suitable population models to increase the relevance of ecotoxicity tests since they incorporate available mechanistic knowledge on the links between responses at the individual level and responses at the population level [12]. Predictions for stressed populations could be achieved provided that dose response relationships are known for the key parameters [13–15]. Moreover, toxicokinetic/toxicodynamic (TK/TD) models, able to link exposure concentration and effects at target organ/system level, can be easily integrated into IBMs and then, the processes and the corresponding effects that lead to toxicity within an organism can be dynamically simulated [16]. A physiologically-based toxicokinetic model [7] and IBM [17] have been recently developed for the zebrafish. However, the IBM developed by Hazlerigg et al. [17] describes life cycle processes without integrated mathematical framework and without integrating some of the main ecological factors driving the population dynamics: photoperiod, water temperature, and food availability [18, 19]. Hence, a more mechanistical approach would offer advantages to improve the realism of such an IBM and to allow, in the future, the integration of toxic effects on physiological processes.

The consequence of the high level of complexity and precision of IBMs is that these models need a large amount of data on the physiological processes involved at the organism-level [20]. The Dynamic Energy-Budget (DEB) theory [21] is a unified mathematical framework composed of models describing mechanistically the acquisition and use of energy to relate growth, maintenance, development, and reproduction to environmental parameters such as food availability and temperature. Then, nesting a DEB model within a population dynamics IBM can be useful when existing models and/or data on organism level physiology are available [2, 22]. A zebrafish DEB model has been developed by Augustine et al. [23] and provides part of the physiological information essential to develop an IBM.

This study aimed to develop a model of the zebrafish population dynamics by coupling the DEB model with an IBM, hereafter referred to as the DEB-IBM. To achieve this, new experimental data on zebrafish growth and reproduction were produced and alternative hypotheses to the existing zebrafish DEB model [23] were used. Then a DEB-IBM integrating the main ecological factors (photoperiod, temperature, and food dynamics) was developed. To perform predictions for stressed populations, the first step was to carefully check the prediction accuracy and reliability of the model under control conditions (e.g. fish population dynamic in the absence of exposure [2]). To this end, we performed a thorough sensitivity analysis of our DEB-IBM and compared the model predictions of the population dynamic in the absence of exposure to field data collected by Spence et al. [24] and Hazlerigg et al. [17].

## Materials and Methods

### New experimental data production

First, length–mass allometry of juveniles was investigated to test the anisomorphic growth hypothesis (change in shape during growth *i*.*e*., **s**urface area grows proportional to volume) proposed by Augustine et al. [23]. Other experiments were designed to produce data to adjust the DEB model for the juveniles (lengths monitored at different feeding regimes), females (fecundity monitored at two different temperatures) and males (female and male lengths monitored at different feeding regimes). All experiments were approved by a local ethics committee: Institut National de l’Environnement Industriel et des Risques (INERIS) “ECOT_11_050_RBU” and “ECOT_11_053_RBu”. Fish were killed by an overdose of MS222.

#### Zebrafish origin, maintenance and culture

Wild type larvae and adult zebrafish (AB strain) originated from our breeding unit (INERIS, Verneuil-en-Halatte, France). Adult zebrafish were maintained in 3.5 L aquaria in a recirculation system (Zebtec, Techniplast, France) at a 14:10 light: dark photoperiod, 27.1 ± 2.0°C. They were allowed to reproduce (2 males for 1 female) in 50L tanks (14:10 light: dark 29°C). Fertilized eggs were collected, disinfected 5 min in water supplemented with 0.1% of commercial bleach (2.6% of sodium hypochlorite). Zebrafish were maintained under semi-static conditions until 21 days and then transferred into 3.5 L aquaria of the recirculation system. For zebrafish culture, fish were fed with commercial diets of different qualities and size to cover all stages of development of fish (Fig. A in S1 Text).

#### Juvenile allometry

To assess length–mass allometry of juveniles, standard length (measured from the tip of the nose to the base of the caudal fin; mm) and wet mass (mg) of 60 juveniles aged from 10 to 50 days post fecundation (dpf) were measured with a precision of 0.1 mm and 0.1 mg, respectively. The relationship between the logarithms of individual length and body masses was then assessed using a linear model.

#### Growth data

Juveniles’ lengths were monitored from 49 to 107 dpf. The juveniles were randomly assigned to three feeding treatments differing in feeding frequencies. Each feeding treatment was replicated three times with n = 15 fish per replicate (15 fish × 3 replicates × 3 treatments = 135 fish in 9 groups/tanks). Fish were fed twice a day with dehydrated food at 6% of their median mass according to the following feeding treatments: (i) each day (Food 1), (ii) two out of three days (Food 0.75), and (iii) every two days (Food 0.5). Fish mass was derived from the length by length–mass allometry relationship and updated at each length measurement. The fish were measured at 49, 56, 63, 70, 77, 91, 104, and 107 dpf.

The measurements of the standard length (mm) were measured by placing the fish onto a Petri dish containing water (less than 5 mm deep). The Petri dish was placed on graph paper and photographed with a digital camera. The digital file was then used to measure the length of the fish to the nearest 0.1 mm using Image J software [25].

Another experiment (following the same protocol) was held with fish groups composed of 30 individuals per group and only two feeding treatments (three replicates per feeding treatment, six fish groups). These individuals were fed following two feeding treatments (6% of their median mass): (i) each day (Food 1), (ii) every two days (Food 0.5). At the end of the experiment, these individuals were sexed by observing their gonad under a binocular microscope.

#### Reproduction data

Two groups of eight females of similar length (from 35 to 43 mm in standard length) were randomly selected. The first group was maintained at 29°C and reproductive outputs were monitored daily for 21 days. The second group was maintained at 26°C and reproductive outputs were monitored daily for 11 days. Each female was held with two males (selected randomly among male of similar length) in a 5 L aquaria supplied continuously with dechlorinated tap water at a flow rate of 5 L.h^−1^. Each evening after the last feeding event, a spawning trap (glass square-shaped beakers with a 2 mm mesh net on the top) was placed in each aquarium. In the morning, the spawning traps were collected and the eggs were counted. Thirty eggs were randomly selected, cleaned, and incubated until 24 hours post-fertilization (1 dpf) at 28°C in autoclaved and aerated aquarium water. After the incubation period, unfertilized eggs were counted. For each group, the spawning at the day 0 was not counted, because this spawning (>500 eggs) corresponded to the eggs cumulated for several days. During the experiment, fish were fed four times a day with SDS 400 (SDS diet) and live brine shrimp *Artemia salina* at a rate of 30% of the fish biomass per day.

### Dynamic Energy Budget model

DEB theory [21] is based on a mathematical description of the uptake and use of energy within an organism, to describe mechanistically the energy flux to physiological process. Moreover, in toxicology, the analysis of (eco)toxicological data through models based on DEB theory (*i*.*e*. DEBTox models) is relevant to mechanistically assess the effect of toxic compounds [26, 27]. According to the DEB theory, energy is taken up from food, assimilated, and stored into reserves. This energy is then dispatched between three main processes: (i) maintenance, (ii) growth, and (iii) maturation/reproduction. The general DEB framework assumes that individuals’ growth follows a von Bertalanffy growth curve provided feeding is *ad libitum* or at constant density [21]. However, as presented by _bookmark2Augustine et al. [23] the zebrafish growth curve could be a sigmoid. The authors used several dataset from Eaton and Farley [28], Bagatoo et al. [29], Schilling [30], Lawrence et al. [31], Best et al. [32], and Gòmez-Requeni et al. [33] to assess their growth curve hypothesis and to estimate parameters of their DEB model. Augustine et al. [23] put forth the hypothesis that the metabolism of larvae accelerates after birth until juvenile stage, *i*.*e*. fish of about 10 mm total body length and with adult fins and pigments [34]. The hypothesis of metabolic acceleration comes with concomitant anisomorphic growth [21]. The metabolic acceleration concept is assumed to impact both the growth curve and the incubation time. Here we propose alternative hypotheses based on feeding limitation and environmental factors. Indeed, as demonstrated on other organisms, a sigmoid growth curve can be explained by a limitation either in the uptake [35, 36] or in the quality of the food [36]. We thus adapted the set of equations from Augustine et al. [23] in order to take into account this new hypothesis. The biological interpretation (and values) of the DEB parameters are listed in Table 1 using standard DEB notations [21]. The compound parameters, which are function of primary parameters, are presented in the supporting information (S2 Text). The primary parameters used in the DEB model were corrected depending on the temperature using a temperature correction function based on the Arrhenius equation (detailed in the supporting information). Thereby, parameters *ν*˙ (mm d^-1^) the energy conductance, $\left\{{\dot{p}}_{Am}\right\}$ (J d^-1^ mm^-2^) the maximum surface area specific assimilation rate, and $\left[{\dot{p}}_{M}\right]$ (J d^-1^ mm ^-3^) the volume specific somatic maintenance costs were corrected. The DEB model including food limitation and fixed size at puberty reads:

$$S_{f}\left(l\right)=\alpha \left[1-{\left(1+\frac{l_{f}^{3}}{l^{3}}\right)}^{-1}\right]$$ (1)

$$\frac{de}{dt}=\frac{{\dot{k}}_{M}g}{l}\left[\left(1-s_{f}\right)f-e\right]$$ (2)

$$\frac{dl}{dt}=r_{B}\left(e-l\right)\;with\;r_{B}=\frac{{\dot{k}}_{M}g}{3\left(e+g\right)}$$ (3)

$$\frac{dR}{dt}=\frac{R_{M}}{1-l_{p}^{3}}\;\left(\frac{g+l}{g+e}el^{2}-l_{p}^{3}\right)$$ (4)

If l < l_b_ then f = 0 and if l < l_p_ then $\frac{dR}{dt}=0$ *sf* is the size-dependant energy ingestion function, *f* (-) is the ratio of the actual ingestion rate divided by the maximal ingestion rate for a given body size, *e* (-) is the scaled reserve density, and *r*_*B*_ (d^-1^) is the von Bertalanffy growth rate. It should be noticed that the birth corresponds to the opening of the mouth. Thus, *Lb* (mm) corresponds to the length at the opening of the mouth. *Lf* (mm) is the length at which the ingestion rate is half the maximum ingestion rate and *Lp* (mm) is the length at puberty. All the physical lengths (*i*.*e*. *L*, *L*_*b*_, *L*_*f*_, and *L*_*p*_) were scaled by the maximal physical length *L*_*inf*_ resulting in the scaled lengths noted *l*, *l*_*b*_, *l*_*f*_, and *l*_*p*_, respectively. Maximal physical length (*L*_*inf*_) is a function of the DEB maximal length and a shape coefficient (Table A in S3 Text). *R* represents the cumulative number of eggs produced, *R*_*M*_ (d^−1^) the maximum reproduction rate, and *g* (-) the energy investment ratio. The DEB model was also adapted for males by assuming that after puberty their food intake is modified by an appetite factor (*f*_*lim*_,-). This factor was calibrated on the experimental data (S1 Text). Thereby, after puberty, the parameter *f* was modified using the following equation:

$$f_{male}=f_{\text{lim}}\times f$$ (5)

The DEB model was calibrated using data from our experiments (Fig. B and C in S1 Text) in addition to a subset of the data used by Augustine et al. [23]. More information on the calibration process is provided in supporting information (S2 Text).

**Table 1:** Parameter abbreviations, values, descriptions and units of the DEB model.

Abbreviation	Value	Description	Unit	Source
*T*_*A*_	3000	Arrhenius temperature	K	[23]
*T*_*R*_	293	Reference temperature	K	[23]
δ	0.20	Shape coefficient ; V^1/3^ = δ.L	-	Fitted
$\left\{{\dot{p}}_{Am}\right\}$	4.72	Maximum area specific assimilation rate	J.d^−1^.mm^−2^	Fitted
$\dot{\nu }$	0.60	Energy conductance	mm.d^−1^	Fitted
*κ*	0.70	Fraction of energy to growth/somatic	-	Fitted
α	0.84	Fraction of food accessible at first feeding	-	Fitted
*l*_*b*_	0.079	Scaled length at the first feeding	-	Fitted
*l*_*p*_	0.58	Scaled length at puberty	-	Fixed
*l*_*f*_	0.163	Length at half maximal food assimilation	-	Fitted
$\left[{\dot{p}}_{M}\right]$	0.44	Volume somatic maintenance costs	J.d^−1^.mm^−3^	Fitted
[*E*_*G*_]	2.35	Cost of synthesis of a unit of structure	J.mm^−3^	Fitted
*E*_0_	1.25	Initial amount of energy in an egg	J	Fitted
*L*_0_	0.25	Initial length	mm	Fixed
*R*_*M*_	406	Maximum number of egg per day	d^−1^	Fitted
*f*_*lim*_	0.92	Male appetite factor limitation	-	Fitted

### Individual-based model of zebrafish population dynamics

The individual based-model (IBM) description follows the ODD (Overview, Design concepts, Details) protocol [37]. Our IBM was developed from the DEB model presented in the previous section. Mating behaviour and survival sub-model was adapted from the model proposed by Hazlerigg et al. [17]. The detailed description of the Design concepts, initialization, Input data and submodel are provided in supporting information (S3 Text). The model was implemented under Netlogo 5.0.4 [38] and the code of the model is provided in supporting information (S4 Text, S1 File and S2 File).

#### Purpose

The zebrafish IBM was developed to extrapolate from individual data measured on organisms under laboratory conditions to biological endpoints convenient to support ecological risk assessment of toxic compounds. To this end, the zebrafish model was developed for a population living in well-suited habitat, *i*.*e*. the abiotic factors (*e*.*g*. temperature, photoperiod) were modelled to represent the natural habitat of this species which are found in North-Eastern India, Bangladesh and Nepal. Field populations were observed near Kolkata [39], thus we selected as reference climatology that of Kolkata, *i*.*e*. a monsoon tropical climate. The zebrafish population that are modelled here inhabits a « median » habitat of the ones described in [24, 39], *i*.*e*. an isolated well-vegetated pond.

#### Entities, state variables, and scales

The model includes two categories of agents: patches and fish agents. Three different patches were modelled: vegetation cover, breeding grounds, and open water patches. Fish agents were sub-divided into four sub-stages: eggs-larvae, juveniles, females, and males. Eggs and larvae (before initiation of feeding) have very similar attributes and processes, and so were therefore grouped into one stage. Eggs-larvae change into juveniles at the onset of exogenous feeding and become females or males at sexual maturity. All agents are characterised by the state variables age (dpf), generation (0 for the initial fish), and survival rate (day^-1^). An eggs-larvae agent represents an entire brood and is characterised by one state variable: the number of surviving eggs. The juvenile, female, and male agents have additional state variables: length (mm), wet body weight (mg), sex, scaled energy density (dimensionless), degree of satiety (dimensionless), and a variable accounting for the variability of the performance between individuals (dimensionless). Female and male agents have each another state variable: the number of eggs (dimensionless) and a state variable characterising the absence/presence and position of the mating territory, respectively.

The fish agents were spatialized (*i*.*e*. agent located in geometrical space) in a two dimensional grid of 900 patches (30 × 30 patches of 20 × 20 cm) representing a typical pond of 36 m^2^. The pond water depth was 0.5 m. Each patch (20 cm × 20 cm × 50 cm) represents the real size of observed zebrafish territories [40]. The time step was 1 day for the IBM (1/10 day for DEB model sub-part).

#### Process overview and scheduling

**Fig 1:**
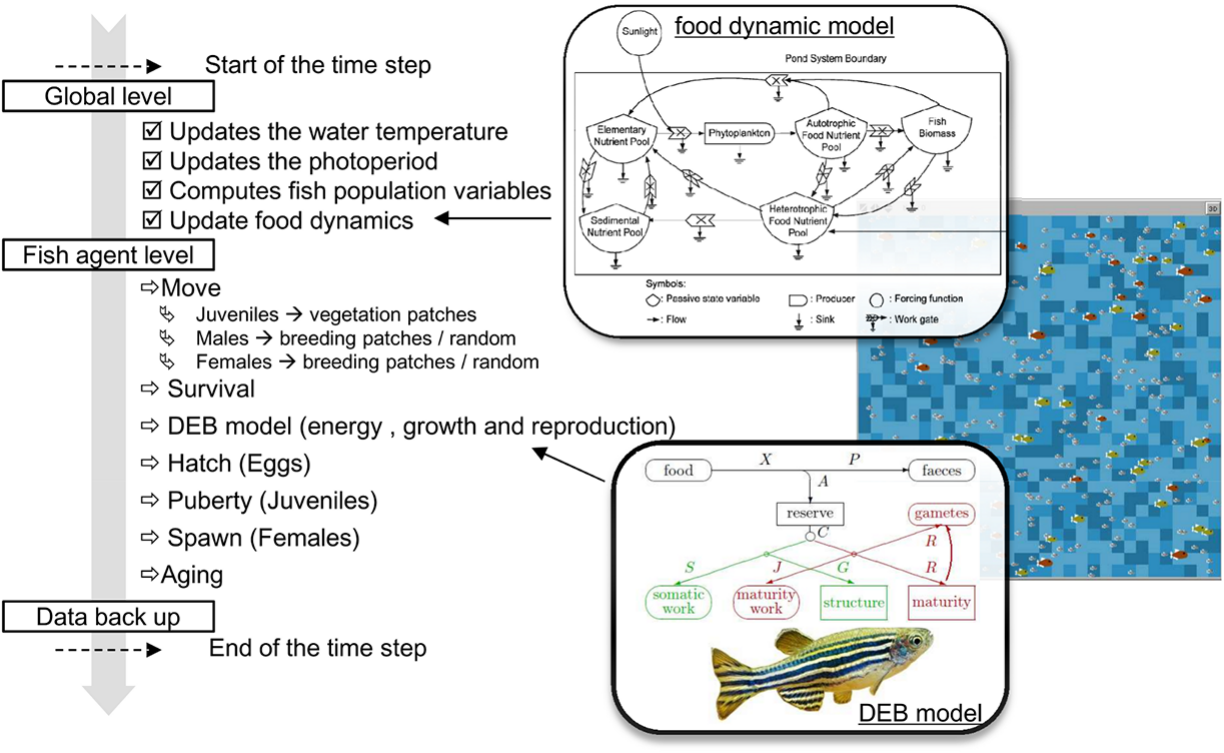
Schema of the schedule of the model of zebrafish population dynamics. Schema of food sub-model was issued from Li and Yakupitiyage [61] and schema of the dynamic energy budget (DEB) model was issued from Kooijman [21]. An arrow before an action denotes that action was realized in random order by fish agents one after another in swift succession.

The schedule is schematized in Fig 1. At each time step, first the environment global variables, *i*.*e*. water temperature, photoperiod, population variables (e.g. fish biomass), and the food dynamic model are updated. Second, fish move each in turn and in random order: juveniles move to find a vegetated patch, males try to establish a territory or move randomly, and females try to find a free males with a territory or move randomly. Then, the fish agents challenge their survival. If alive, fish agents grow. After that, if the conditions are met, eggs-larvae agents can hatch, juvenile agents can undergo puberty, and female agents can spawn. At the end of the time step, the simulation results are backed up.

### Statistical methods

#### Sensitivity analysis

The sensitivity analysis orders the inputs by importance, identifying the main contributors to the variation in the model outcomes. Two different methods were used for the DEB model and IBM analysis to keep a reasonable time of computing. Sensitivity analysis of the DEB model was performed using the variance-based Sobol method [41, 42]. This sensitivity analysis method is a global and model independent method (nonlinear and non-monotonic functions and models can be handled) and is based on variance decomposition (quantifying the amount of variance that each parameter contributes to the unconditional variance of the model output). This approach allows identification of the contribution of each parameter (First order Sobol’ sensitivity index) and the interactions between parameters (Total Sobol’ sensitivity index). Sensitivity analysis of the IBM was performed using Morris method [43]. This method identifies the few important factors at a limited cost of simulations. It also gives information on the global contribution of each parameter and the linearity of the effects or the interaction with other parameters. Details of these analyses are provided in (S5 Text).

#### Model parameterisation

DEB model parameters calibrations were performed using GNU MCSim software [44], which is a program for the statistical analysis of Bayesian hierarchical models by Markov Chain Monte Carlo (MCMC). Details are provided in the supporting information (S2 Text). The parameter values of the IBM were picked-up from the literature (Tables 2 and 3) and three parameters were fitted (no *a priori* information): π_a_, π_c,_ and F_in_ (survival parameters and food input during the monsoon; Table 3). Two different datasets were used to calibrate and assess predictability of the DEB-IBM model: data collected by Hazlerigg et al. [17] and data collected by Spence et al. [24], respectively. These authors have measured length on a random sample of fish captured from a sub-part of the monitored populations (field sampling methods were ineffective in catching smaller individuals). These data were compared with the fish length distribution predicted by the model for the sub-part of the population considered (after the simulation of three years to stabilise the population; 1000 simulations). Hence, we assume that the probability to be sampled for a given fish was related to the frequency of the fish length in the sub-population. Calibration was done using a genetic algorithm provided by the open-source software BehaviorSearch [45] with a generational population-model and a mutation-rate, crossover-rate, population-size, and tournament-size equal to 0.01, 0.7, 50 and 3, respectively. The distance was the sum of squares of the difference between the length distribution predicted by the model (after 1110 days, fish with a length > 18.4 mm) and the length distributions observed by Hazlerigg et al. [17] (length distributions had similar bin of 1 mm).

**Table 2:** Parameter abbreviations, values, descriptions and units of the food sub-model.

Abbreviation	Value	Description	Unit	Source
G2Kcal	1.88	Coefficient of energy by mass of zebrafish	Kcal.g^-1^	[23]
s	21.08	Proportionality of food nutrient quantity to fish biomass	-	[61]
h_n_	0.2	Half saturation nitrogen (N)	mg.L^-1^	[61]
h_p_	0.02	Half saturation phosphorus (P)	mg.L^-1^	[61]
K_n_	0.01	N-fixation coefficient of phytoplankton	g.kcal^-1^.day^-1^	[61]
K_fn_	0.017	N content of fish	g.kcal^-1^	[61]
K_an_	0.0224	N content of phytoplankton	g.kcal^-1^	[61]
K_hn_	0.0192	N content of heterotrophic components	g.kcal^-1^	[61]
K_sn_	0.003	Release coefficient of N in sediment	day^-1^	[61]
K_nl_	0.17	coefficient of inorganic N loss to air	day^-1^	[61]
K_ap_	0.001	P content of phytoplankton	g.kcal^-1^	[61]
K_hp_	0.001	P content of heterotrophic components	g.kcal^-1^	[61]
K_pr_	0.0006	Release coefficient of P in sediment	m.day^-1^	[61]
K_ps_	0.28	Coefficient of inorganic P sedimentation to sediment	m.day^-1^	[61]
K_s_	0.14	Coefficient of heterotrophic food sedimentation	day^-1^	[61]
K_d_	0.12	Coefficient of heterotrophic food decomposition	day^-1^	[61]
λ_max_	1.6	Maximum growth coefficient for phytoplankton growth	day^-1^	[61]
K_r_	0.1	Coefficient of phytoplankton respiration	day^-1^	[61]
K_ml_	0.6	Coefficient of autotrophic food entering heterotrophic food pool	day^-1^	[61]
Ir	6.547	Reference solar radiation	10^6^ cal.m^-2^.day^-1^	[61]
Ir_a_	0.000017	Light extinct coefficients for autotrophic components	pond.Kcal^-1^	[61]
Ir_b_	0.000015	Light extinct coefficients for heterotrophic components	pond.Kcal^-1^	[61]
T_opta_	30	Optimal temperature for phytoplankton growth	°C	[61]
k_T1_	0.004	Effects of temperature below Topta on growth	°C^-2^	[61]
K_T2_	0.008	Effects of temperature above Topta on growth	°C^-2^	[61]

**Table 3:** Parameter abbreviations, values, descriptions and units of the IBM.

Abbreviation	Value	Description	Unit	Reference
W_d_	0.5	Water depth of the pond	m	[24, 39]
W_v_	18	Water volume of the pond	m^3^	[24, 39]
Np_b_	207	Number of patches with breeding grounds	-	[17]
Np_v_	207	Number of patches with vegetation cover	-	[17]
Nb_j_	300	Number of juveniles in the initial population	-	[17]
Nb_m_	35	Number of males in the initial population	-	[17]
Nb_f_	35	Number of females in the initial population	-	[17]
End	1110	Simulation duration	d	This study
σ	0.235	Inter-individual variability of energy acquisition	-	This study
P.τ	12	Photoperiod threshold to the onset/offset of the reproduction	h	This study
T.τ	22.5	Temperature threshold to the onset/offset of the reproduction	°C	[62]
H_0.5_	24	Female density inducing 50% reduction of hte hacthing rate	g.mm^-3^	[63]
H_max_	0.89	Optimal hatching rate	-	[64–66]
R.τ	263	Number limit of eggs to spawn	-	This study
SR_μ_	50	Sex-ratio genetic mean	-	[67]
SR_σ_	23.1	Sex-ratio genetic variability	-	[67]
SR_a_	-0.0496	Slope of the effect of temperature on sex-ratio	°C^-1^	[68]
SR_b_	27.9	Effect of temperature on sex-ratio	-	[68]
π_a_	0.0292	Natural mortality probability	d^-1^	Fitted on population data
π_b_	- 0.3820	Allometric scaling factor	-	[17]
π_c_	0.9576	Density-independent mortality constant	d^-1^	[17]
π_d_	0.0089	Density-dependent mortality constant	-	Fitted on population data
π_p_	0.0250	Daily egg predation probability	d^-1^	[17]
π_e_	2.839e^-06^	Effect of age on mortality	d^-1^	[69]
π_g_	550	Age threshold of mortality due to aging	d	[69]
Fl_m_	0.91	Male appetite modified by male puberty	-	This study
F_inputs_	0.0142	inputs during the monsoon period	-	Fitted on population data
H_a_	60.9	age at birth in degree.days	°C. d^-1^	[34]
H_b_	10.3	Threshold of the degree.days	°C	[34]
W_a_	3.205	Slope of the relationship log(W)/Log(L)	-	This study
W_b_	-5.193	intercept of the relationship relation log(W)/Log(L)	-	This study

#### Statistical analysis

Data were analysed using ANOVA or RM-ANOVA. When not normal or not homoscedastic, data were transformed using the Box Cox method [46]. Scheffe’s post hoc test was used to compare two groups with a significant limit of p < 0.05. Statistical analyses were performed using R statistical environment [47].

## Results

### New experimental data

All the newly generated data on juvenile allometry, growth, and reproduction are presented in S1 Text. A linear model was adjusted between the mass logarithm and the length logarithm. The slope and intercept of the linear model between the mass logarithm and the length logarithm were 3.22 with a 95% confidence interval of (2.97; 3.46) and -5.24 with a 95% confidence interval of (-5.83; -4.65), respectively. Hence, the slope of the linear model did not significantly differ from 3 (organism mass is proportional to the cubic length in case of isomorphic allometry; Fig. F in S1 Text). Significant effects of feeding regime and time were observed in the group of 15 and 30 fish. For the second group (30 fish), a significant sexual dimorphism on the length was observed at 107 dpf.

Inter-spawn intervals were 1.59 ± 1.10 days and 1.23 ± 0.76 days for the females held at 26°C and 29°C, respectively (Fig. D in S1 Text). The interval was equal to 24h for 71% and 85% of the intervals of the females monitored at 26°C and 29°C, respectively. The temperature did not impact the mean number of eggs per spawning (263 ± 104.2 eggs). A significant effect of female/male couple on the percentage of viable eggs at 24h post fecundation was observed. The median percentage of viable eggs at 24 hours post fecundation per females varied from 40 to 100% (Fig. E in S1 Text).

### DEB and IBM sensitivity analysis

**Fig 2:**
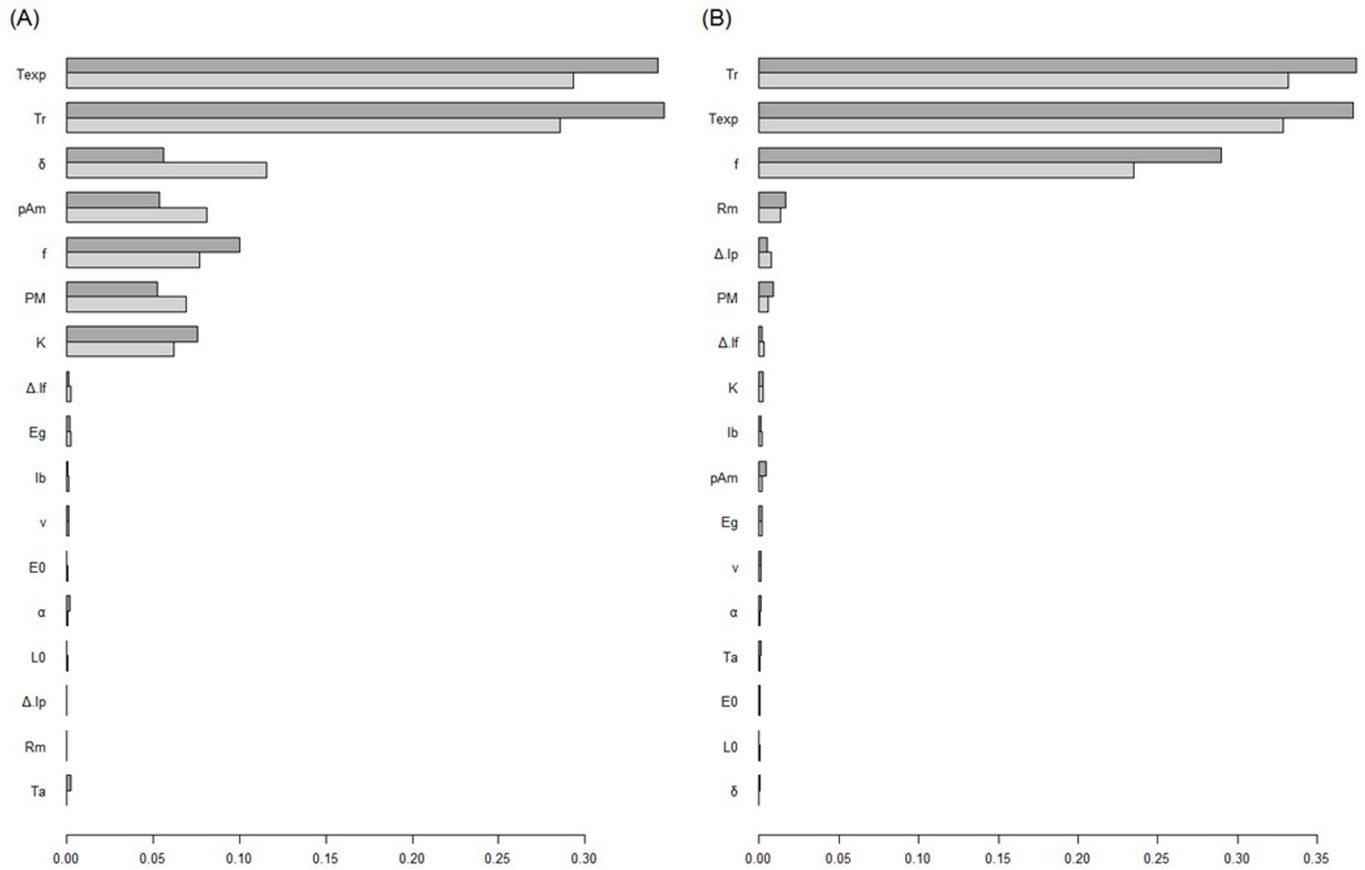
Mean of the DEB model sensitivity analysis results for length equations (A) and reproduction equations (B). Parameters are ordered according to the Sobol’s first order indices (light gray). Dark gray indices are the Sobol’s total indices. First order (Si) and total Sobol’ sensitivity indices (STi) were estimated at 25, 50, 75, 100, 200, and 400 dpf for length prediction and at 100, 200, and 400 dpf for the reproduction prediction.

**Fig 3:**
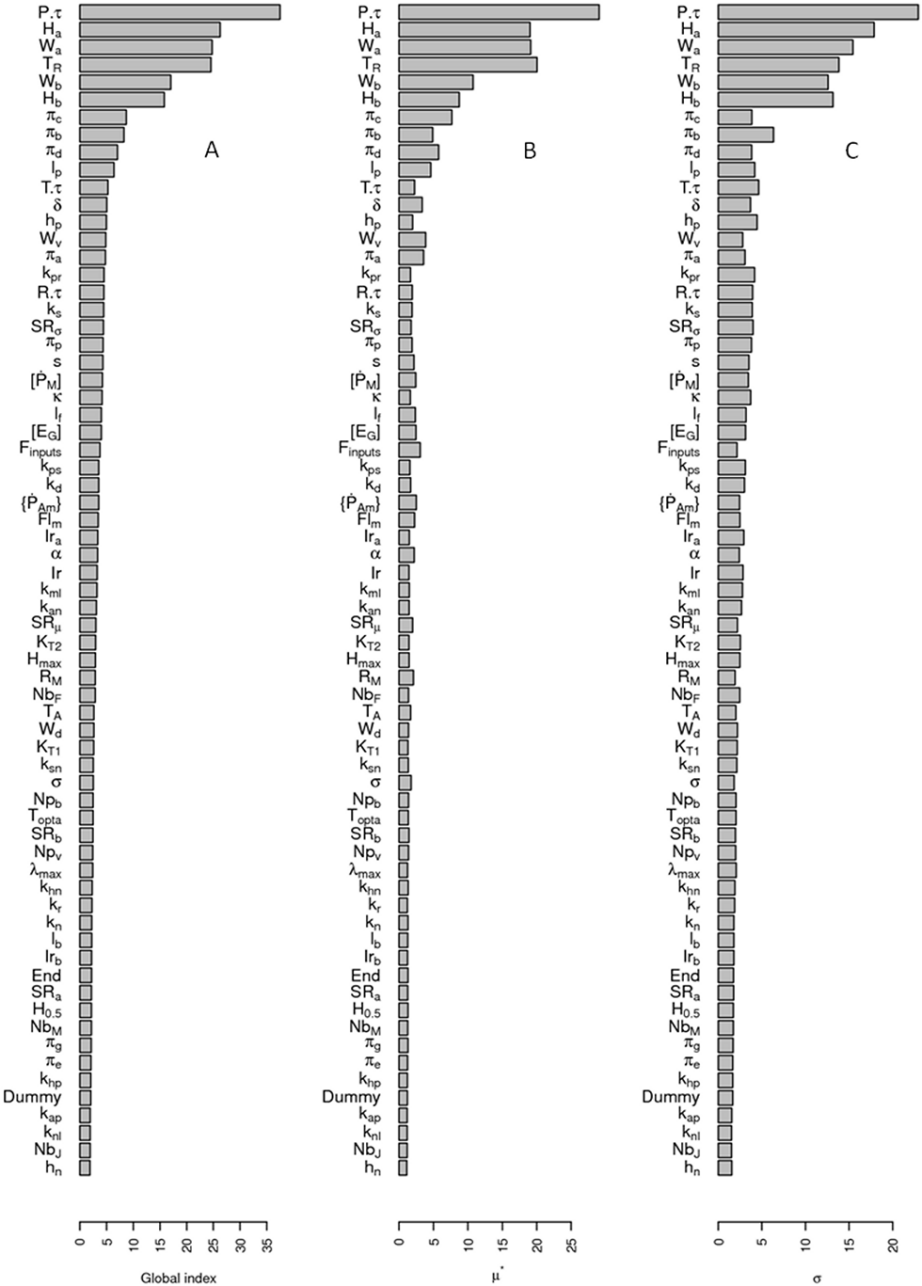
Morris’ global index (A), mean of the elementary effects (B) and standard deviation of the elementary effects (C) determined for the IBM of the zebrafish population dynamics. Presented indices were the mean of the indices determined for the total number of fish, the frequency of adult/juvenile, the mean length of adults and juveniles at 1095, 1156, 1217, 1277, 1339, 1400 days from the beginning of the simulations. Sensitivity analysis indices were calculated on the mean of 30 model repetitions for each parameter combination. "Dummy" is a parameter with no influence on the model outputs. The other parameters are presented Tables 1, 2 and 3.

Sensitivity analysis performed on the DEB model (Fig 2 and S5 Text) showed that the main contributors to the DEB model output variations for the growth part of the model were the experimental and reference temperature, the shape coefficient, the maximum surface area specific assimilation rate, the actual ingestion rate divided by the maximal ingestion rate for a body size, the volume specific somatic maintenance costs, and the fraction of energy mobilised from the reserves which is allocated to growth and somatic maintenance for the growth part of the model. The experimental temperature, the reference temperature, the actual ingestion rate divided by the maximal ingestion rate for a body size, and the maximal reproduction rate were the main contributors to the reproduction part of the DEB model. As expected, these parameters of the DEB model were also in the most influent parameters in the DEB-IBM sensitivity analysis (Fig 3).

The DEB-IBM sensitivity analysis showed that all the important parameters presented both high mean and standard deviation of the Morris' elementary effects (Fig 3 and Fig. B and C in S5 Text). This means that these parameters present non-linear effect or strong interaction with other parameters. Among the most influent parameters, a great part of them are linked to ecological factors. For instance, the photoperiod parameter (P.τ), parameters driving effects of temperature (T_r_, T.τ), and several parameters of the food sub-model (h_p_, K_pr_, and K_s_) have a high impact on the model outputs (Fig 3). In particular, the photoperiod parameter (P.τ) presents a large μ* and σ for total abundance and juvenile frequency at 1095 days (May, just after the beginning of the reproduction). This denotes a strong regulation of the recruitment of juveniles by photoperiod, more than by the water temperature in our model. As expected, the parameters driving the mortality were important (π_c_, π_b_, π_d_ and π_a_; Fig 3). These parameters impact mainly the total abundance, the male and female frequency and the mean length (Fig. B in S5 Text). Interestingly, the initial parameters of the simulations (Nb_J_, Nb_F_, Nb_M_, and End) have a very low impact on the global index (Fig 3).

### DEB and IBM model calibration

**Fig 4:**
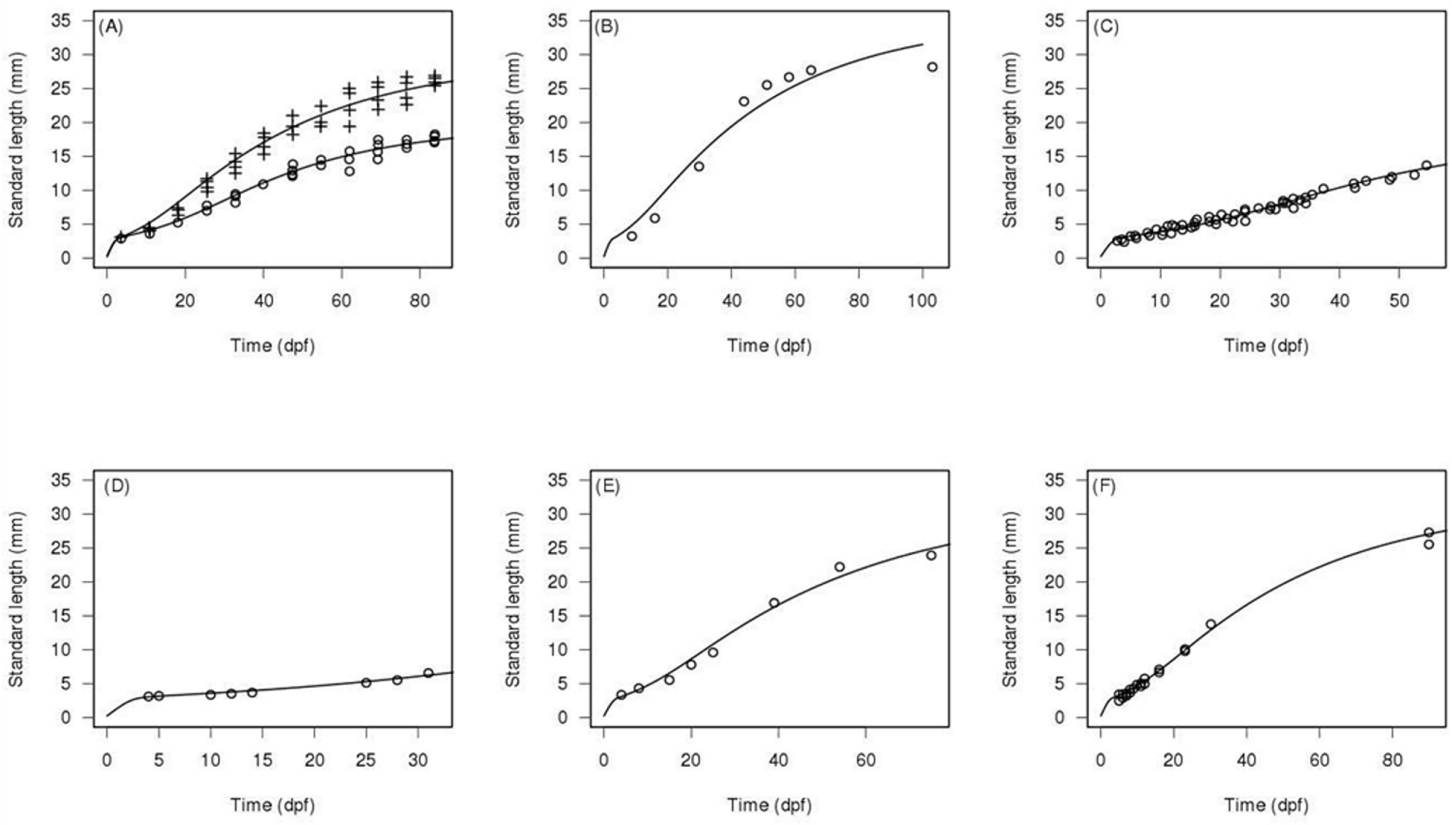
DEB model simulations against observed length for fish from various literature experiments. All lengths are presented as standard length (mm). Circles and crosses represent observations, lines represent model predictions. Panel A represents dataset from Lawrence et al. [31], experimental temperature (T_exp_) of 28.5°C, estimated ingestion level (*f*) of 0.54 (circles) and *f* = 0.80 (crosses). Panel B represents dataset from Gòmez-Requeni et al. [33], T_exp_ = 28°C, *f* = 0.96. Panel C represents dataset from Schilling [30], T_exp_ = 28°C, *f* = 0.52. Panel D represents dataset from Bagatto et al. [29], T_exp_ = 25°C, *f* = 0.47. Panel E represents dataset from Eaton and Farley [28], T_exp_ = 25.5°C, *f* = 0.86. Panel F represents dataset from Best et al. [32], T_exp_ = 25°C, *f* = 0.87

**Fig 5:**
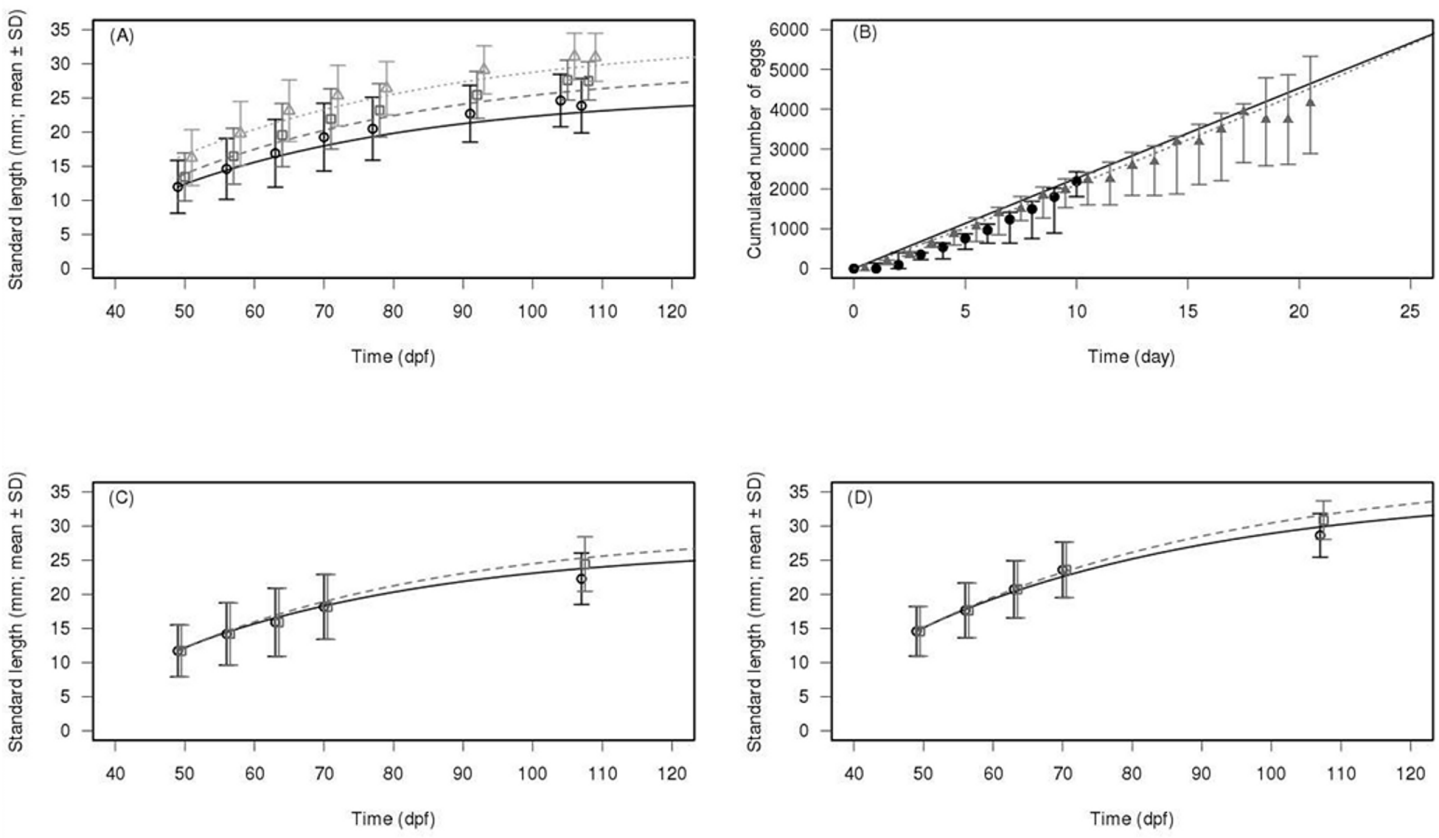
DEB model simulations compared to the experimental data produced in this study. All lengths are presented as standard length (mm). Points represent observations (mean ± SD for length data), lines represent model predictions. (A) Length data, experimental temperature (T_exp_) of 27°C, estimated ingestion level (*f*) of 0.70 (circles), 0.81 (squares), and 0.93 (triangles). (B) Median of the cumulated number of eggs, T_exp_ = 26°C, *f* = 0.48 (points) and T_exp_ = 29°C, *f* = 0.93 (triangles); Error bars represent the first and third quartiles (C) Model predictions against observed length data for males (black circles and solid line) and females (grey squares and dashed line). T_exp_ = 27°C, *f* = 0.76. (D) Model predictions against observed length data for males (black circles and solid line) and females (grey squares and dashed line). T_exp_ = 27°C, *f* = 0.99.

The DEB calibration was performed using three independent MCMC chains. The Gelman and Rubin convergence statistics were inferior to 1.2 for all parameters [48, 49]. This indicates that the three chains converged to the same solution. Our DEB model provided a relevant fit of both the growth and the reproduction data for several datasets at several temperatures from both new experiment and the literature (Figs 4 and 5). The *f* parameter, linked to environmental feeding conditions was fitted for each experiment. Prior and posterior distributions of the parameters are presented in Table A in S2 Text except for the *f* parameter which is presented with the relevant figures.

**Fig 6:**
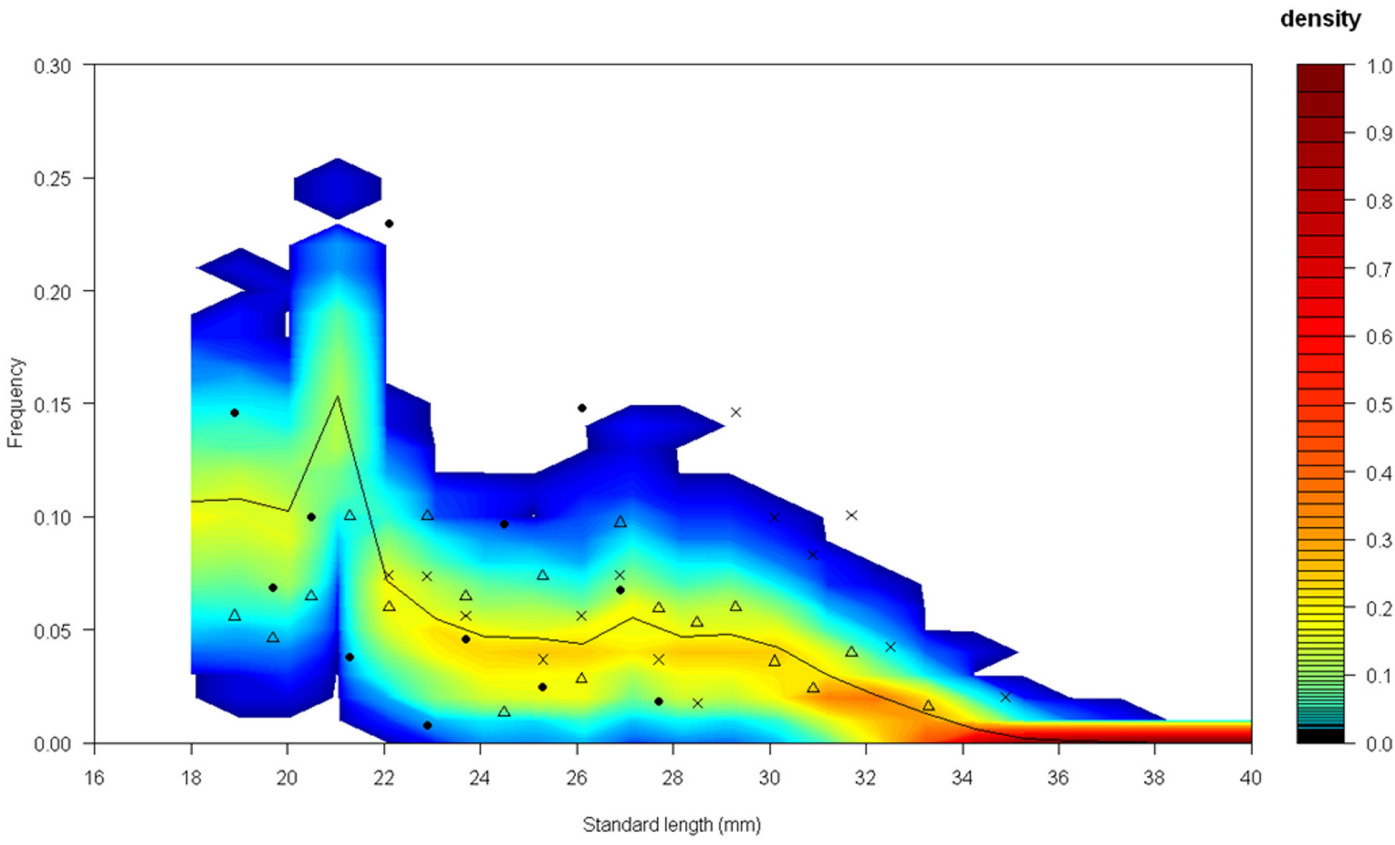
Probabilistic distributions of the length frequency predicted by the model length compared to frequency distributions observed in [17]. Circles, crosses and triangles represent the length frequency distribution of the three observed populations. Full lines represent the median length frequency distribution of the simulated populations. Colour level represents the frequency of simulated populations (n = 1,000) having a given percentage of individuals for a given class length. Frequency inferior to < 1e-05 was represented in white. The length class was one millimetre. The frequencies in the populations of the length class frequencies was calculated using class of 0.01.

**Fig 7:**
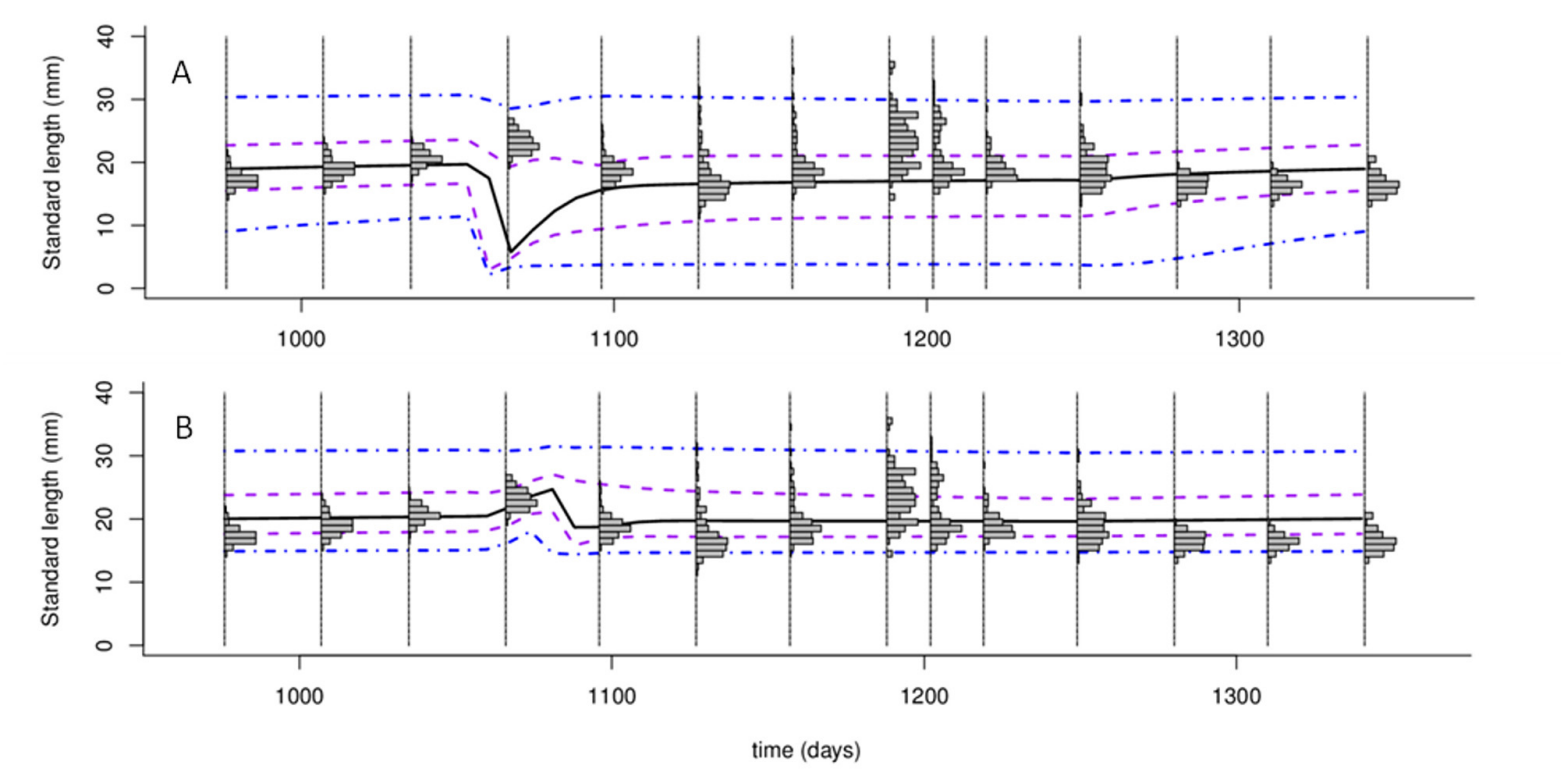
Zebrafish length distributions predicted compared to the length observed by Spence et al. [24]. (A) Predicted length distributions of all fish. (B) Predicted length distributions of fish > 15 mm. Black full lines represent the median length, purple dotted lines represent the first and third quartile of the fish length, and blue lines represent the limits of 95% of the fish length. Bar plot represent the distribution of the length of the fish sampled by Spence et al. [24] (sample of 120 fish).

**Fig 8:**
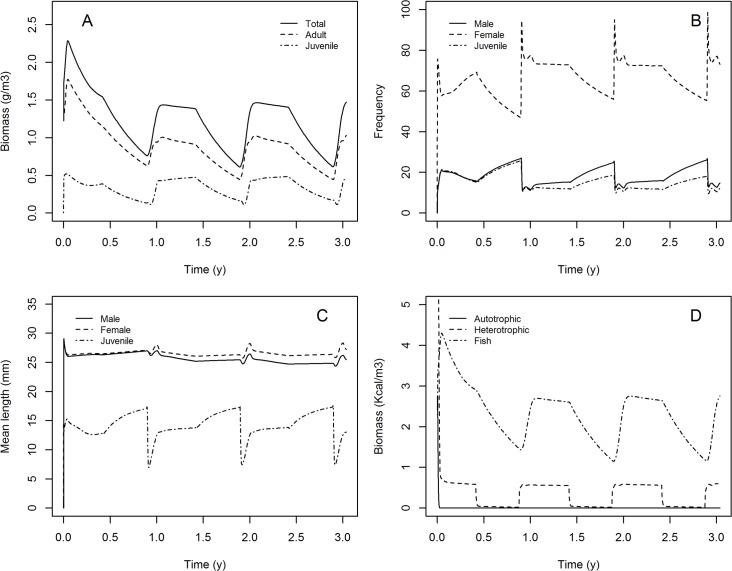
Zebrafish population dynamics predicted through three years. (A) zebrafish biomass, (B) frequencies of the different fish stages (excluding eggs-larvae), (C) mean length of the different zebrafish stages, and (D) food dynamic state variables.

To fit the three IBM parameters (survival parameters and food input during the monsoon), three independent fitting to the data collected by Hazlerigg et al. [17] were done and converged to the same solution. The adjusted values of π_a_, π_c_, and F_in_ are presented in Table 3. Fig 6 presents the model prediction compared to the data collected by Hazlerigg et al. [17]. The model provided an accurate prediction of these data, outside of the frequencies of the larger individuals (from 29 to 33 mm) in one population monitored (*i*.*e*., this population presented a higher calibration distance with the best parameterisation). In this population, larger individuals were more frequent than other length class, contrary to the two other observed populations and the simulated populations. The data reported by Spence et al. [24] were compared to model predictions without any parameter fitting (Fig 7A and 7B). Observation done by Spence et al. [24] are censored due to the field sampling methods. The length above which the data were censored is supposed to be 15 mm (fifth percentile of the lengths observed by Spence et al. [24]). Thereby, if individuals smaller than 15 mm were excluded from the model predictions (Fig 7B), the evolution through the year of the length distribution as predicted by our model is in good agreement with the length distribution of the sub-sample of the population monitored (Fig 7B). The proportion of individuals excluded from the simulated populations varies from a minimum of 17% during non-breeding period to a maximum of 91% during the breeding period (Fig 8B).

All variables of the model presented an annual cycle with a clear difference between the non-breeding period and the period favourable for reproduction (monsoon period) (Fig 8). The juvenile biomass represented a small part of the biomass (panel A), whereas in abundance, the juveniles represented a large part of the population during the breeding-period (panel B). Fish biomass was larger than autotrophic and heterotrophic biomass (panel D).

## Discussion

One of the main challenges to ecotoxicology and ecological risk assessment of chemicals is to develop tools and strategies to characterize environmental hazard and risk assessment of chemicals for aquatic environment. To this end, we developed a model of the zebrafish population dynamics that combined DEB and IBM models to extrapolate from ecotoxicological data measured on organisms in laboratory conditions to biological endpoints convenient to support ecological risk assessment of toxic compounds. For that purpose, new experimental data on zebrafish growth and reproduction were produced and used to propose alternative hypotheses to the zebrafish DEB model published by Augustine et al. [23]. Then a DEB-IBM couple model integrating the main ecological factors (photoperiod, temperature, and food dynamics) was developed.

Our data on the relationship between masses and lengths of juveniles (from the hatching length to near 15 mm) do not support the hypothesis of anisomorphic growth. Hence, we proposed a zebrafish DEB model based on an alternative hypothesis: a limitation of the food/energy intake linked to the length at the hatching. Such food limitation intake modification relative to standard DEB models has already been used for other organisms [35, 50].

Two other modifications of the model were done to reduce the computation costs of the whole DEB-IBM model. The maturity variables were removed by assuming a constant length at birth and puberty. For the length at birth, as the sensitivity analysis showed a very low impact of the size at birth on the population dynamics, the possible deviations due to this simplification compared to full DEB model are clearly insignificant. For the length at puberty, DEB model predicted that this physiological parameter could vary of about 10% for large variation of food availability [23]. Nevertheless, this limited variation due to food, in addition of the overall small impact of this parameter on the population dynamics showed by the sensitivity analysis (see Fig 5), suggest that this simplification is not of high impact on the population dynamic in our DEB-IBM model.

The second modification concerned the incubation time. An alternative hypothesis was proposed to predict the incubation time, the embryo dynamics have been excluded by using an equation based on the Kimmel et al. [34] empiric formula. According to the data collected by Kimmel et al. [34], for the zebrafish, the incubation time is mostly driven by the local temperature.

The incubation time and maturity variables simplifications of the model were done to reduce the computation costs of the whole DEB-IBM model while still fits experimental data very well. As the model predictions were accurate, it can be assumed that the modifications present relevant alternative hypothesis to the previous published zebrafish DEB model [23].

Another modification of the DEB food intake was introduced to take into account the sexual dimorphism. Indeed, an appetite factor lowered the male’s food intake. This modification was introduced in order to take into account the male zebrafish behaviour modification after the puberty and then the lower male maximal length [24]. Indeed, our data suggested that the energy demand of the males is lower than that of females (males eat less than females), as the males were generally smaller even if both sexes were fed in excess. This was also observed in several other teleost fish [51, 52]. The appetite factor modification is not so far from the type A metabolic acceleration described by Kooijman [53]. The author describes a shift in the surface area-specific assimilation capacity between males and females of the same species at some stage of the development. Some other studies addressing DEB modelling for both males and females in marine species have opted to calibrate several DEB model parameters independently on both males and females [54] or to focus on independent *κ* parameter for both sexes [55] to explain the sexual dimorphism.

The length at puberty was not fitted in our model as we considered that available data did not allow a relevant fit of this parameter. Therefore, this parameter was fixed according to literature.

The sensitivity analysis performed on the zebrafish DEB model showed that experimental conditions are the main contributor to the model output variation. Hence, a particular care should be paid to experimental conditions such as feeding and temperature across the whole life cycle of the organisms in order to fully analyse experimental data with modelling tools, and in particular, ecotoxicological data.

Few data were found to assess the predictability of our model. In addition, these data are censored due to the field sampling methods. Hazlerigg et al. [17] noted that field sampling methods were ineffective in catching smaller individuals. Indeed, less than 5% of fish sampled by Spence et al. [24] measured less than 15 mm. Hence, to compare the predicted length distribution to these reported by Spence et al. [24], the fish smaller than 15 mm have to be excluded. Consequently, length distributions were estimated on a sub-sample of the entire population and at some time points, a large proportion of the population was not observed. These two points resulted in a relative high uncertainty on the length class frequencies estimated and then, could explain some divergences between the model predictions and the observations. In particular, the relative high frequency of the larger individuals (from 29 to 33 mm) in one population observed in [17] contradicted usual fish population length distributions. Indeed, usually, older (larger) individuals are less frequent than younger fish [13].

Annual variation of the different state variables that were predicted by the food dynamic sub-model was classically observed in aquatic systems with similar characteristics to the one we modelled in this study [56–58]. Indeed, fish have a strong impact on the auto and heterotrophic compartments (top-down control). Over the entire year, in our food dynamic sub-model, balance between the nutriment input and output is supposed null to ensure sustainability of the ecosystem. Hence, the nutriment loss is compensated by inputs during the monsoon period, which is described as a high food availability period [56]. Actually, during the monsoon the extent of freshwater habitats is maximal with wide variation [56] and runoff conveys a large amount of heterotrophic particles and nutriments from terrestrial sources to aquatic ecosystem [59].

When DEB-IBM model predictions were compared to the observations, the predicted population dynamics seems likely and coherent with ecological knowledge on fish population dynamics. Nevertheless, some processes could be upgraded to improve the mechanistic realism of our model. First, it should be noticed that a lack of precise environmental information regarding the photoperiod and daily water temperature in the natural range of the zebrafish imposed to re-build scenario for these two inputs (linear interpolation or model prediction). However, as sensitivity analysis showed, the parameters which are related to these factors are highly influent on the model outputs (DEB model and IBM). Therefore, more precise information on these environmental factors and on their impacts on the zebrafish population dynamics should be acquired.

Contrary to the model developed by Hazlerigg et al. [17], we did not integrate the reduction of mortality rate of juveniles according to their location. Actually, there is no carrying capacity for the vegetated patch, so all juveniles stay in vegetated patches and which led to a global modification of the relationship between length and survival probability. Thus, it does not seem necessary to take into account the impact of the location on the juvenile mortality. Survival process is globally the most uncertain in our model. Indeed, very few data are available to model and calibrate this process. However, the parameters driving the mortality were identified as important in the sensitivity analysis, except the parameters driving the senescence. This is in agreement with the observations in wild populations in which senescence was not observed [56]. It is likely that fish die in natural populations before the senescence process occurs. Globally, more detailed data on the entire wild population structure and dynamics should allow us to more precisely model the survival probability. The relative feeding level is determined assuming that fish feeding has a paralleling mechanism, *i*.*e*. the food resource is shared equally to all the fish (proportionally to their need). Nevertheless, the food resources are probably different between juveniles and adults, and dominant individuals attempt to monopolise a food source [56]. In addition, as no mortality would occur in adult female zebrafish after 21 days of starvation [60] and as any other data are available, no explicit modelling of the starvation effect on mortality was integrated. This assumption is realistic, as many fish species evolved the capability to endure prolonged food shortages due to seasonal change in food availability [19]. However, further experiments are needed to understand the mechanism of the food partition and starvation, and to improve this part of our model.

Finally, the annual cycle of zebrafish growth rates should be investigated. Currently, the energy fluxes modelled by the DEB model are identical between the non-breeding period (winter) and the period with active reproduction (spring and summer) and energy use for reproduction is supposed dissipated in winter. No observation on this process was found for the zebrafish. In other fish species, it was observed that non-reproducing females grew more than the reproductively active females [19]. New data on growth obtained during photoperiodic offset of the reproduction should allow us to propose more precise modelling of the non-breeding period.

## Conclusion

A DEB-IBM model has been successfully developed for the zebrafish taking into account the main environmental parameters for the fish population dynamics. The DEB-IBM model was calibrated, deeply analysed, and the predictions were compared to the few published zebrafish population observations. Globally, when the model predictions were compared to the observations, the predicted population dynamics seems highly probable. While, our zebrafish DEB-IBM model can still be improved by acquiring new experimental data on the most uncertain processes (e.g. survival or feeding), it can already serve to predict impact of compounds at the population level and serve as a basis for future works aiming at assessing the ecological impact of chemicals acting on the endocrine system of fish. Hence, the next step will be to compare our model predictions on the impacts of some compounds at the population level with the observed data to test its predictability performances.

## Supporting Information

S1 File Model input file. Input file of temperature and photoperiod scenario. This file is called by the model as "InputDataFromMay.txt".
(TXT)

S2 File ZIP file archive of the Netlogo model file. (ZIP)

S1 Text New experimental data. (DOCX)

S2 Text DEB model calibration. (DOCX)

S3 Text Models details. (DOCX)

S4 Text Netlogo code of the zebrafish IBM. (DOCX)

S5 Text Sensitivity analysis. (DOCX)
