# Supplementary material for: An Individual-Based Model of Zebrafish Population Dynamics Accounting for Energy Dynamics
Source: PLoS One. 2015 May 4;10(5):e0125841. doi: 10.1371/journal.pone.0125841 (PMC4418570; doi:10.1371/journal.pone.0125841)
Supplement: S1 Text — (DOCX) [file pone.0125841.s003.docx]

Appendix S1. New experimental data

## Food regimes used at different developmental stages of zebrafish.

**Figure A**. Food regimes used at different developmental stages of zebrafish. For dried diets, the amount of food was approximately 6% of the body mass. In adult, this percentage increases to 30% to support increase of energy expenditures when they reproduce. From 10 dpf, the food was supplemented daily with live brine shrimps *Artemia salina*. Protogen (Hooby) was obtained from Europrix, (France); SeraMicron (Sera) from Europrix (France), SDS from Scientific diet Foods (France) and artemia from our local culture. dpf = day post-fertilization.

## Growth data with 15 individuals per group.


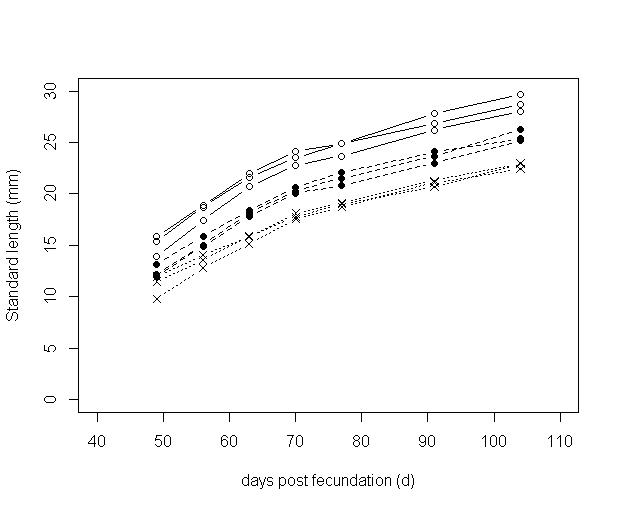


**Figure B**. Median standard length of 15 fish fed each day (circles), two out of three days (dark points), and every two days (crosses) with 6% of their wet mass of dehydrated food (3 replicates by treatment).

**Table A**. Final effective of the fish group (n initial = 15) fed each day (Food 1), two out of three days (Food 0.75) and every two days (Food 0.5).

|  | **Food 1** | **Food 0.75** | **Food 0.5** |
| --- | --- | --- | --- |
| M | 3, 3, 5 | 3, 4, 6 | 5, 3, 3 |
| F | 5, 5, 4 | 8, 8, 8 | 3, 6, 2 |
| N tot | 15, 12, 15 | 14, 13, 15 | 15, 14, 13 |

## Growth data with 30 individuals per group.


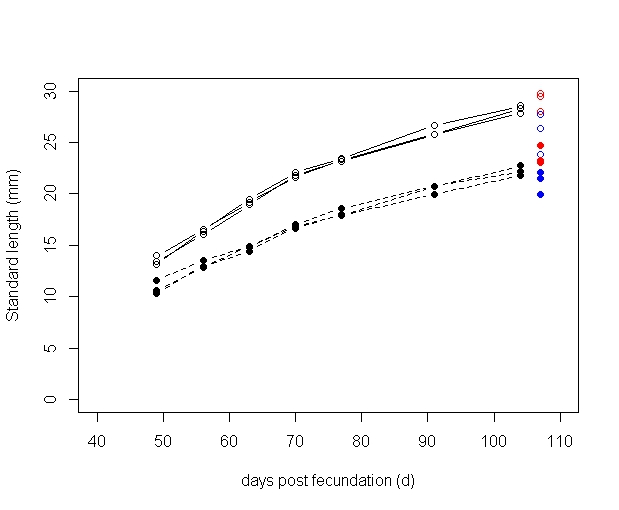


**Figure C.** Median standard length of 30 fish fed each day (circles) and every two days (points) with 6% of their wet mass of dehydrated food (3 replicates by treatment). At the last time of measurement, red and blue points represent the females and the males, respectively.

Red and blue points represent the median standard length measured at the end of the experiment in females and males, respectively. The phenotypic sex was identified by visual observation of their gonads.

**Table B.** Final effective of the fish group (n initial = 30) fed each day (Food 1), two out of three days (Food 0.75) and every two days (Food 0.5).

|  | Food 1 | Food 0.5 |
| --- | --- | --- |
| M | 3, 8, 12 | 13, 12, 5 |
| F | 18, 15, 15 | 10, 9, 20 |
| ? | 8, 6, 0 | 5, 6, 2 |
| N Tot | 29, 29, 27 | 28, 27, 27 |

## Reproduction data


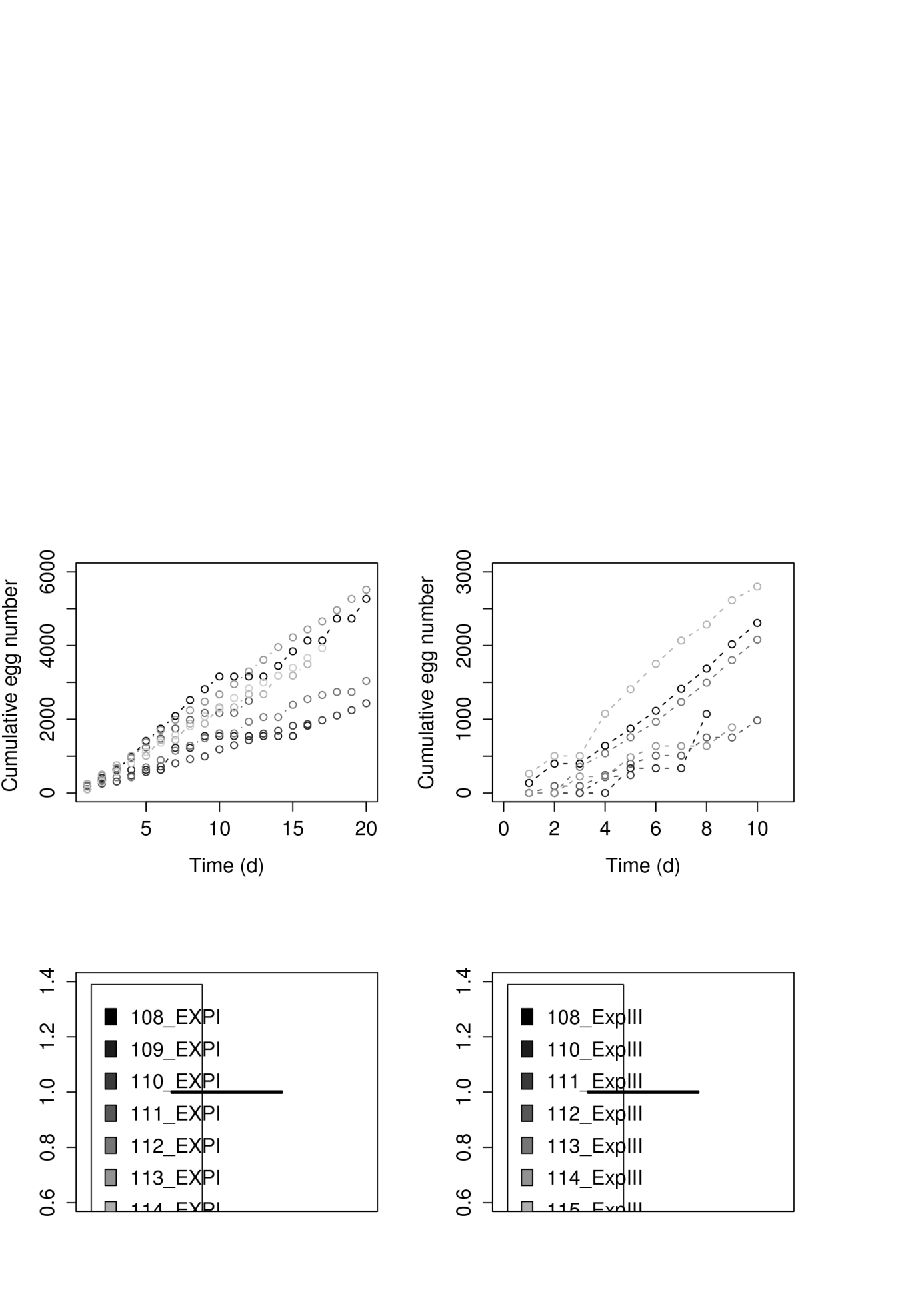


B

A

**Figure D.** Cumulative egg number per female recorded for 21 days at 29 °C (A) and seven days at 26 °C (B). Each series of data represents a female that was allowed to spawn every day. The sex ratio was 1 female for 2 males. Two females did not spawn at 26°C during the seven days and were excluded from the analysis. While spawning was continuous for most individuals, 4 (out of 8) and 2 (out of 6) females stopped to spawn before the end of the experiment without any obvious explication, respectively at 29°C and 26°C.


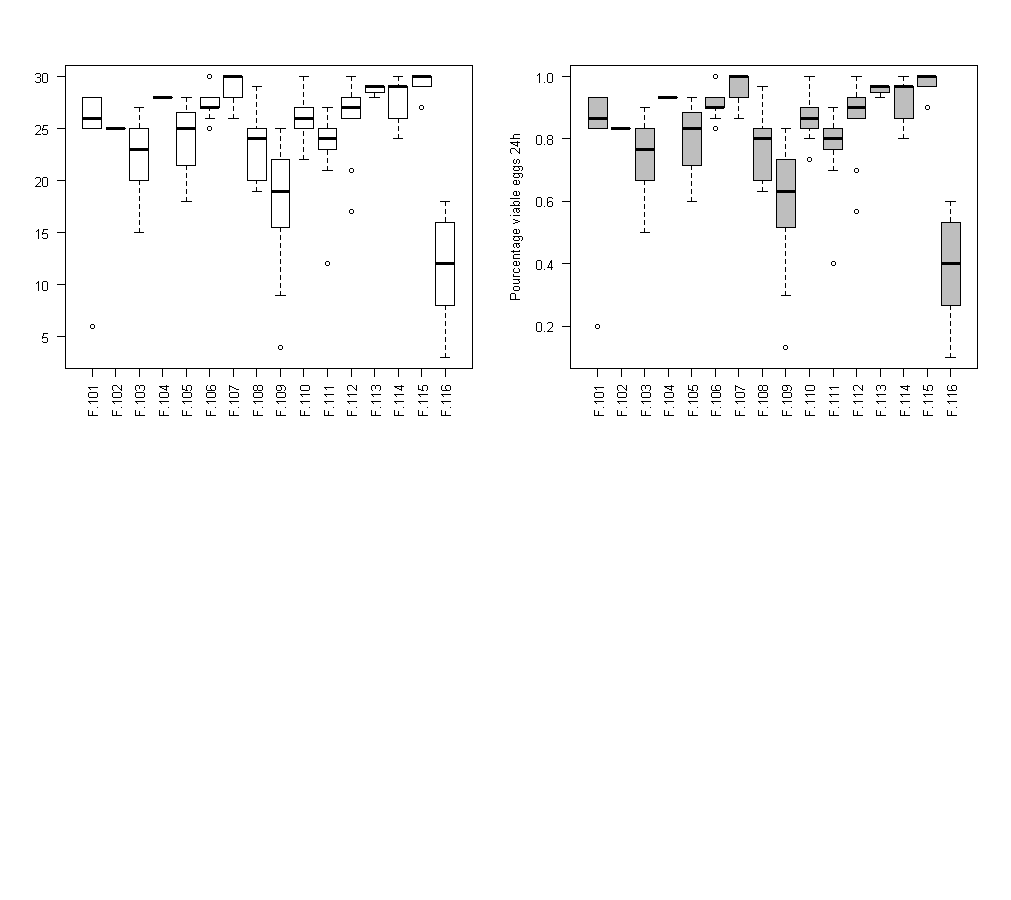


Percentage of viable eggs at 24 hpf

**Figure E.** Percentage of viable eggs at 24 hours post fecundation (hpf) according to the female/males groups.

## Body mass and standard length relationship


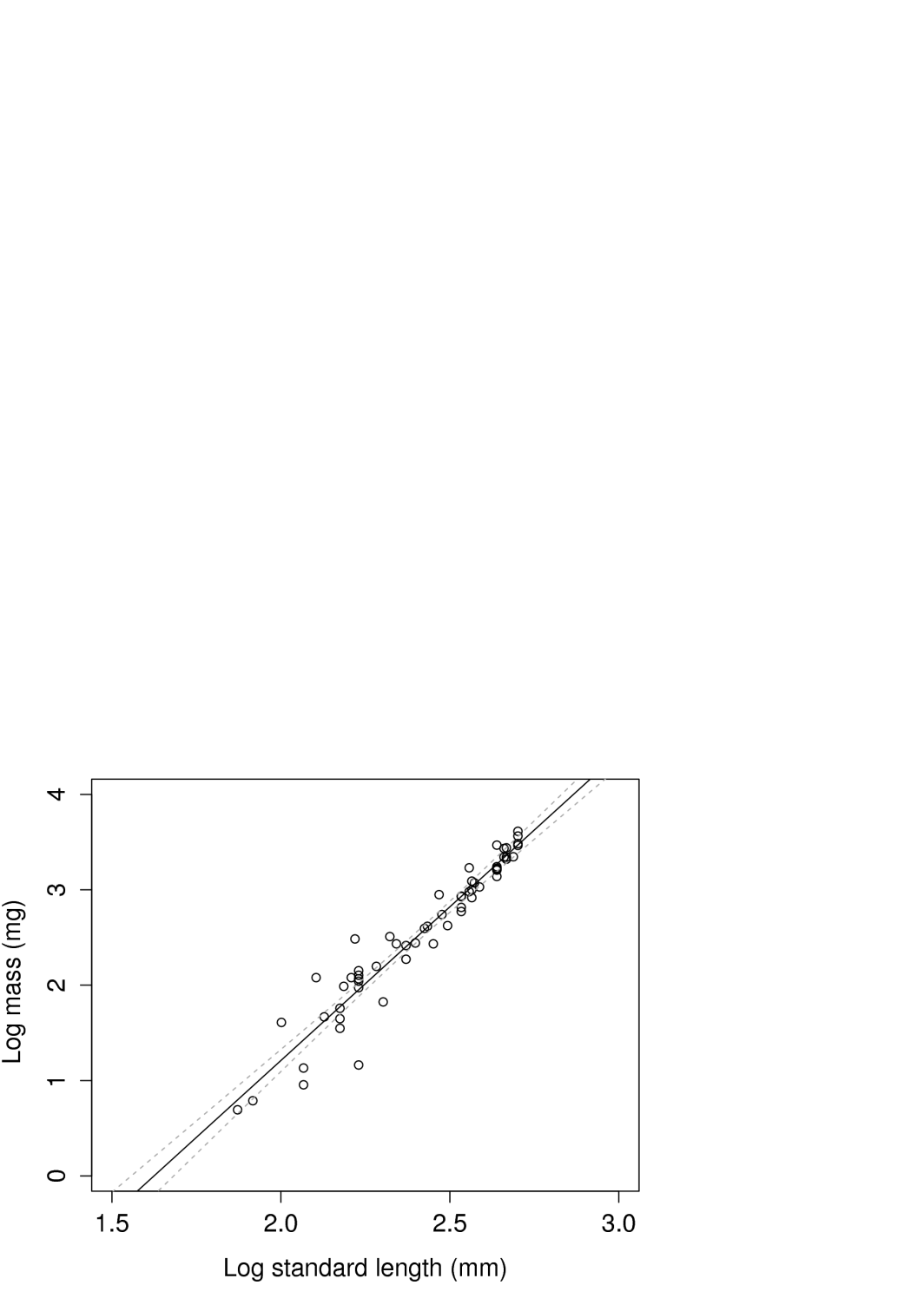


**Figure F.** Linear relationship between the logarithm of individual body mass (mg) and the standard length (mm). The slope and intercept of the linear model are 3.22 [2.97 – 3.46] and -5.24 [-5.83 - -4.65]. Model was fitted on data from 60 fish measuring from 6.5 mm to 14.9 mm (R^2^=0.92). Grey stippled lines represent the model confidence interval.
