# Supplementary material for: An Individual-Based Model of Zebrafish Population Dynamics Accounting for Energy Dynamics
Source: PLoS One. 2015 May 4;10(5):e0125841. doi: 10.1371/journal.pone.0125841 (PMC4418570; doi:10.1371/journal.pone.0125841)
Supplement: S2 Text — (DOCX) [file pone.0125841.s004.docx]

Appendix S2. DEB model calibration

Parameters estimations were performed using the GNU MCSim software (Bois and Maszle, 1997), which is a program for the statistical analysis of Bayesian hierarchical models by Markov Chain Monte Carlo (MCMC) (Plummer, 2003). The initial parameters’ distributions were chosen according to the literature (Augustine et al., 2011) and authors’ expert opinion (Table S4). A positive truncated normal distributions was chosen for biological parameters and a coefficient of variation of 30% was applied to these values except for the initial energy reserve in an egg (*E_0_*) for witch a coefficient of variation of 10% was applied. Parameters for which less information was available were distributed according to a non-informative uniform distribution. 1,000,000 iterations were performed on three independent MCMC chains. Chains’ convergences were assessed using the Gelman and Rubin (1992) convergence statistic modified by Brooks and Gelman (1998). The Arrhenius temperature and the reference temperature parameters were fixed according to the literature (Augustine et al., 2011). The *f* parameter was fitted for every experiment. As available reproduction data did not allow a relevant fit for *l_p_*, this parameter was fixed to an arbitrary biologically relevant value according to the literature (Eaton and Farley, 1974; Lawrence et al., 2012). The data observation process is supposed distributed according to a normal distribution.

**Table A**. Prior and posterior distributions of the parameters of the DEB model.

| Parameter | Prior | Posterior (mean*±*SD) |
| --- | --- | --- |
| *TA* (K) | 3000 | 3000 |
| *TR* (K) | 293 | 293 |
| δ (−) | *N*(0.132, 0.04) T [0*,* 1] | 0.20 ± 0.01 |
| α (−) | *U* [0*,* 1] | 0.84 ± 0.05 |
| $\left\{ \dot{p}_{Am} \right\}$ (J d*−*1 mm*−*2) | *N* (2*.*5*,* 0.75) T ]0*,* +*∞*] | 4.72 ± 0.32 |
| *ν* (mm d*−*1) | *N* (0*.*278*,* 0.084) T ]0*,* +*∞*] | 0.60 ± 0.06 |
| κ (−) | *N*(0.4366, 0.13) T ]0*,* +*∞*] | 0.70 ± 0.06 |
| *f* (−) | *U* [0*,* 1] | For each experiment |
| lb (−) | *N*(0.075, 0.0022) T [0*,* 1] | 0.079 ± 0.002 |
| $\Delta l_{f}$ (-) | *N* (0*.*225, 0.045) T [0*,* 1] | 0.084 ± 0.01 |
| lp (−) | 0.58 | 0.58 |
| [*p*˙*M* ] (J d*−*1 mm*−*3) | *N* (0*.*5009*,* 0.15) T ]0*,* +*∞*] | 0.44 ± 0.05 |
| [*EG*] (J mm*−*3) | *N* (4.652*,* 1.36) T ]0*,* +*∞*] | 2.35 ± 0.35 |
| *RM* (# h*−*1) | *N* (240*,* 72) T ]0*,* +*∞*] | 406.0 ± 39 |
| *E_0_* (J) | *N*(1.67, 0.167) T ]0*,* +*∞*] | 1.25 ± 0.17 |
| $f_{Lim}$ (-) | *U* [0*,* 1] | 0.92 ± 0.02 |

The normal distribution of mean *µ* and standard deviation *σ* is noted *N* (*µ, σ*), and the uniform distribution between *a* and *b* is noted *U* [*a, b*]. T]*c, d*] denotes an interval censoring between *c* (excluded) and *d*. *lb, lf ,* and *lp* are scaled length parameters. l_p_ was fixed according to the literature.

Augustine, S., Gagnaire, B., Floriani, M., Adam-Guillermin, C. and Kooijman, S.A.L.M., 2011. Developmental energetics of zebrafish, Danio rerio. pp. 275-283.

Bois, F.Y. and Maszle, D., 1997. MCSim: a simulation program. Journal of Statistical Software, 2(9):<http://www.stat.ucla.edu/journals/jss/v02/i09> (software available online at [www.toxi.ineris.fr)](http://www.toxi.ineris.fr)).

Brooks, S.P. and Gelman, A., 1998. General Methods for Monitoring Convergence of Iterative Simulations. pp. 434-455.

Eaton, R.C. and Farley, R.D., 1974. Spawning cycle and egg production of zebrafish, Brachydanio rerio, in the laboratory. Copeia, 1:195–204.

Gelman, A. and Rubin, D.B., 1992. Inference from Iterative Simulation Using Multiple Sequences. pp. 457-472.

Lawrence, C., Adatto, I., Best, J., James, A. and Maloney, K., 2012. Generation time of zebrafish (Danio rerio) and medakas (Oryzias latipes) housed. Lab animal, 41:158-165.

Plummer, M., 2003. JAGS: A program for analysis of Bayesian graphical models using Gibbs sampling. Proceedings of the 3rd International Workshop on Distributed Statistical Computing (DSC 2003). March, pp. 20-22.
