# Supplementary material for: An Individual-Based Model of Zebrafish Population Dynamics Accounting for Energy Dynamics
Source: PLoS One. 2015 May 4;10(5):e0125841. doi: 10.1371/journal.pone.0125841 (PMC4418570; doi:10.1371/journal.pone.0125841)
Supplement: S3 Text — (DOCX) [file pone.0125841.s005.docx]

Appendix S3. Models details

## Dynamic energy budget details

**Table A.** Compound parameters of the DEB model

| **Compound parameter** | **Equation** | **Description** |
| --- | --- | --- |
| $\left[ E_{M} \right]$ (J mm^-3^) | $\frac{\left\{ \dot{p}_{Am} \right\}}{\dot{\nu}}$ | Maximum reserve density |
| *g* (-) | $\frac{\left[ E_{G} \right]}{\kappa\left[ E_{M} \right]}$ | Energy investment ratio |
| $\dot{k}_{M}$ (d^-1^) | $\frac{\left[ \dot{p}_{M} \right]}{\left[ E_{G} \right]}$ | Somatic maintenance rate |
| *L_inf_*  (mm) | $\frac{\dot{\nu}}{\dot{k}_{M}g}\times\frac{1}{\delta}$ | Maximum physical length |
| *l_f_*  (-) | $l_{b}+\Delta l_{f}$ | Scaled length at which the ingestion rate is half the maximum ingestion rate |

$\left\{ \dot{p}_{Am} \right\}$ (J d^-1^ mm^-2^) represents the maximum surface area specific assimilation rate, $\dot{\nu}$ (mm d^-1^) is the energy conductance, $\left[ E_{G} \right]$ (J mm^-3^) is the cost of synthesis of a unit of structure, $\kappa$ (-) is the fraction of energy mobilised from reserve which is allocated to growth and somatic maintenance, $\left[ \dot{p}_{m} \right]$ (J d^-1^) is the volume specific somatic maintenance costs, *δ* (-) is the shape coefficient, $l_{b}$ (-) is the scaled length at birth,$\Delta l_{f}$ and $\Delta l_{p}$ represent the change in scaled length to reach *l_f._*

DEB parameters were adjusted in function of the experimental temperature ($T_{Exp}$) using a temperature correction function ($T_{c}$) based on the Arrhenius temperature ($T_{A}$) and the reference temperature ($T_{R}$):

$T_{c}=exp(\frac{T_{A}}{T_{R}}-\frac{T_{A}}{T_{Exp}})$ Eq. (1)

$\dot{\nu}=\dot{\nu}_{ref}\times T_{c}$ Eq. (2)

$\left\{ \dot{p}_{Am} \right\}=\left\{ \dot{p}_{Am} \right\}_{ref}\times T_{c}$ Eq. (3)

$\left[ \dot{p}_{M} \right]=\left[ \dot{p}_{M} \right]_{ref}\times T_{c}$ Eq. (4)

A parameter value followed by “ref” indicates that the value is for the reference temperature.

## Food sub-model details

Food dynamics is predicted using a simplified version of the model by Li and Yakupitiyage (2003) designed to predict nutrient dynamics of semi-intensive pond fish culture. The simplified model predicts the dynamics of seven compartments: phosphorus in water (P_w_, g.pond^-1^), in sediment (P_s_, g.pond^-1^), nitrogen in water (N_w_, g.pond^-1^), in sediment (N_s_, g.pond^-1^), autotrophic food (F_A_, Kcal.pond^-1^), heterotrophic food (F_H_, Kcal.pond^-1^), and fish biomass (FB, Kcal.pond^-1^). In our model, fish biomass is calculated at each time step by the IBM as the sum of the fish mass in g converted in Kcal (in zebrafish, 2.17 Kcal.g^-1^(Augustine et al., 2011) (Augustine et al., 2011)) and fish eat only heterotrophic food. Autotrophic food is supposed to represent a negligible caloric incoming for the zebrafish (5.9% of autotrophic food in mean stomach content, Spence et al. 2007). Fish biomass dynamic is the result of the fish anabolism (F_G_), the fish catabolism (F_C_) and fish mortality (FB_d_, kcal.day^-1^.pond^-1^). Fish anabolism, fish catabolism and fish dead biomass are predicted by the DEB-IBM.

The relative feeding level of the fish i (f_i_) is calculated assuming that fish feeding has a paralleling mechanism:

$f_{i,t}=\varphi_{i}\times f_{h,t}$ Eq. (5)

$f_{h,t} = 1-e^{-s\times\left( \frac{F_{H}}{FB} \right)^{2.2}}$ Eq. (6)

${Ec}_{i,t} = f_{i,t}\times\left\{ \dot{p}_{Am} \right\}\times V^{2/3} \times\psi= f_{i,t}\times\left\{ \dot{p}_{Am} \right\}\times\frac{L}{\delta}\times\psi$ Eq. (7)

$HFc=\sum_{i=1}^{n} {Ec}_{i,t}$ Eq. (8)

Where Ec_i,t_ is the energy consumed by the fish i at the time t in kcal. *φ_i_* is the inter-individual variability related to dominance in foraging, ψ is the conversion coefficient of the energy in J to Kcal ( 1 Kcal = 4186.8 J) and HFc is the heterotrophic food consumed by the fish (kcal.day^-1^.pond^-1^)

Photosynthesis, by phytoplankton and macrophytes, is a unique source of autotrophic food (AFG, kcal.day^-1^.pond^-1^), while losses of autotrophic food include phytoplankton respiration (AFR, kcal.day^-1^.pond^-1^) and mortality/ harvesting by secondary producers (AFM,  kcal.day^-1^.pond^-1^*)*:

$\frac{dF_{A}}{dt}=AFG-AFR-AFM$ Eq. (9)

$AFG={F_{A}\times\lambda}_{max}\times\left( f_{n,p}\times f_{i}\times f_{Ta} \right)$ Eq. (10)

$AFR=F_{A}\times k_{r}$ Eq. (11)

$AFM=F_{A}\times k_{ml}$ Eq. (12)

λ_max_ is the maximum growth rate for phytoplankton growth (day^-1^). f(N,P), f (I) and f(Ta) are limiting functions of elementary nutrients, solar radiation and water temperature to phytoplankton growth (dimensionless, see Li and Yakupitiyage, 2003). k_r_ is a coefficient of phytoplankton respiration (day^-1^), K_ml_ is a coefficient of autotrophic food entering heterotrophic food pool due to phytoplankton mortality and harvest by secondary producers (day^-1^). F_A_ is supposed to be greater than 1×10^-3^ Kcal.pond^-1^ to avoid definitive extinction.

Heterotrophic food refers to all living and non-living heterotrophic components of particulate organic matters in a pond that can be grazed by zebrafish. Sources of heterotrophic food in ponds include autotrophic food mortality (AFM, kcal.day^-1^.pond^-1^), fish faecal wastes (FW, kcal.day^-1^.pond^-1^) and biomass of dead fish (eggs, larvae, juveniles and adults; FB_d_) at a time step converted in Kcal. Heterotrophic food decline is determined by decomposition (HFD, kcal.day^-1^.pond^-1^), sedimentation (HFS, kcal.day^-1^.pond^-1^), grazing by fish (HFC) and the respiration of living heterotrophic components.

$\frac{dF_{H}}{dt}={FB}_{d}+ AFM-HFC-HFS-HFD$ Eq. (13)

$HFS=F_{H}\times k_{s}$ Eq. (14)

$HFD=F_{H}\times k_{d}$ Eq. (15)

Compared to the model of Li and Yakupitiyage (2003), the impact of water quality on decomposition of heterotrophic particles (HFD) was not integrated. Hence, dissolved oxygen is assumed to be ever superior to 4 mg/L. K_maxa_ is the maximum assimilation coefficient of the fish (day^-1^). k_s_ and k_d_ are coefficients of heterotrophic food sedimentation and decomposition (day^-1^).

Nitrogen compartment dynamic:

$\frac{dN_{w}}{dt}= AFR\times k_{an}+HFD\times k_{hn}+N_{s}\times k_{sn}-N_{AF} -N_{eva}$ Eq. (16)

$N_{AF}= AFG\times k_{an}-N_{fix}$ Eq. (17)

$N_{eva}=-N_{w}\times k_{nl} and N_{fix}= k_{n}\times F_{A}{\times e}^{-k_{n}{INC}^{2}}$ Eq. (18)

$\frac{dN_{s}}{dt}= HFS\times k_{hn} - N_{s}\times k_{sn}$ Eq. (19)

N_fix_ is the nitrogen fixation rate, which is influenced by phytoplankton quantity and inorganic concentration nitrogen. k_fn_, k_an_ and k_hn_ are nitrogen contents of fish tissue, phytoplankton and heterotrophic components (g. kcal^-1^). k_sn_ is a release coefficient of nitrogen in sediment (day^-1^), k_nl_ is a coefficient (day^-1^) of inorganic nitrogen loss into air (N_eva_).

Phosphorus compartment dynamics:

$\frac{dP_{w}}{dt}= AFR\times k_{ap}+HFD\times k_{hp}+P_{s}\times\frac{Kpr}{W_{d}}- P_{w}\times\frac{Kps}{W_{d}}- AFG\times k_{ap}$ Eq. (20)

$\frac{dP_{s}}{dt}= HFS\times k_{hp}+P_{w} \times\frac{K_{ps}}{dw}-P_{s}\times\frac{K_{pr}}{dw}$ Eq. (21)

k_fp_, k_ap_ and k_hp_ are phosphorus contents of fish tissue, phytoplankton and heterotrophic components (g. kcal^-1^). k_pr_ and K_ps_ are the release and catch coefficient of phosphorus in sediment (m.day^-1^) and W_d_ is water depth (m).

On the entire year, balance between the nutriment input and output is supposed null. Hence, the nutriment and heterotrophic food loss are compensated by inputs during the monsoon period (high food availability during the monsoon months; Spence et al. 2008). Hence, a percentage of the initial amount of N and P was injected all the day in the pond during this period of the year (*F_inputs_* = 1.13 %).

## Individual based model details

### Design concepts

*Basic principles.* Five main factors govern the population dynamics of the teleost fish, including zebrafish: photoperiod, water temperature, food availability, predation, and density-dependence (Godin, 1997; Wootton, 1998). Thus, developing a model to predict population recovery and/or resilience and then performing realistic ecological risk assessments of a pollutant on population dynamics needs to integrate these factors (Galic et al., 2012). The photoperiod and water temperature were integrated in our model as inputs (cf. Input data section). Specific sub-models were integrated to predict food availability and impacts of predation on zebrafish survival. Finally, density-dependence impacts on fish mortality and female fecundity were incorporated as proposed in the model of Hazlerigg et al. (2014).

*Emergence*. Population dynamics and fish length distributions emerge from the behaviours, inter-individual interactions and interactions, with the environment of the fish agents.

*Adaptation and objectives*. Dominant females and males prioritise mating with dominant (larger) individual to maximize their fitness and those of their offspring.

*Learning and prediction*. Fish agents do not learn, express cognitive action or predict the future conditions.

*Sensing*. Individuals sense the number and biomass of the other fish globally over the whole pond. These population variables were supposed to be convenient proxies of the pheromones concentration which modulates physiological processes (*e.g.* female fecundity). Competition for reproductive territories implies that adults have a global perception of the pond (choose free patches among all the pond patches) and of the lengths of the other fish (dominance relation).

*Interaction.* Fish agents interact indirectly through density-dependent processes mediated by fish or female biomasses. Direct competition between adult agents takes place to search and occupy breeding ground patches. Competition for breeding ground patches is mediated by the lengths of the individuals.

*Stochasticity*. The model is intrinsically stochastic: fish survival is a stochastic process. Moreover, inter-individual variability of the process and attributes of the agents was integrated in the model (cf. inter-individual variability in the sub-model section). The last source of variability in our model was the definition of the initial population, which was drawn at the beginning of each simulation.

*Collective*. Fish agents did not form or belong to aggregations that affect the individuals.

*Observation.* Model predictions were compared to data collected by Spence et al. (2007) and by Hazlerigg et al. (2014). Spence et al. (2007) measured the standard length on a random sample of 120 fish collected each month from January to December 2005 using a fine mesh. Hazlerigg et al. (2014) sampled fish using a fine mesh from three populations of wild zebrafish in Bangladesh in May 2009 and they have collected 482 wild zebrafish with a length > 18.4 mm (23 mm in total length, field sampling methods were ineffective in catching smaller individuals).

### Initialization

For all the analyses (sensitivity analysis, calibration, or predictions), the initial population was setup as the wild populations of zebrafish observed directly in Bangladesh by Hazlerigg et al. (2014) : 35 adult males, 35 adult females, 300 juveniles. As the observations were made in May, the simulation started in May. Initial lengths of the Juveniles were drawn between the birth length (Lb) and the puberty length (Lp). Initial lengths of the adults were drawn between the puberty length and the maximal length. In these two cases, the length distributions were uniform, as any information on the length distribution was provided. Age of the fish was defined according to the scaled length (length / maximal length) with a maximal age fixed at 287 days post fecundation (Hazlerigg et al., 2014). The initial numbers of eggs for females and energy density were randomly drawn from 0 to the maximal value with uniform probability. Other initial attribute values of initial individuals were set as for all other individuals. Hence, a random initial population was drawn at the beginning of each simulation, representing a possible real population as observed by Hazlerigg et al. (2014).

The pond patches were setup with 23% (207 patches; Np_b_) vegetation cover, 23% (207 patches; Np_v_) breeding grounds, and 54% (486 patches) open water. This habitat setup corresponds to those found in 8 natural ponds harbouring zebrafish during a survey undertaken in Bangladesh (Hazlerigg et al., 2014).

The global state variable of the food sub-model were initialized at the values observed in the pond before fertilization (first observation time) by Li and Yakupitiyage (2003). Nitrogen, phosphorus, autotrophic, and heterotrophic compartments were initialized at 0.97 g.m^-3^, 0.01 g.m^-3^, 2.5 Kcal.m^-3^, and 18.4 Kcal.m^-3^, respectively. Amounts of nitrogen and phosphorus were supposed equal between the water and sediment at the beginning of the simulation.

### Input data

The first input is the photoperiod recorded at Kolkata (Bangladesh) in 2013 (Source timeandate.com, model predictions with a maximal error of about 1 min). Daily mean water temperature is also integrated as an input. The daily water temperature was predicted from a linear interpolation from discrete data recorded by Bala and Hasan (1999) in ponds in Bangladesh to continuous time*.*

### Submodels

Our IBM was developed from the DEB model presented in the previous section.

**Food dynamics**. Zebrafish are euryphagous omnivores possessing no teeth, no true stomach and a long intestine with a large absorption area (Ulloa et al., 2012). Their natural diet consists primarily of zooplankton and aquatic stages of insect species (Spence et al., 2007). The food dynamics is predicted using a simplified version of the model of Li and Yakupitiyage (2003) designed to predict nutrient dynamics of semi-intensive pond fish culture. The simplified model predicts the dynamics of seven state variables: elementary nutrients (phosphorus and nitrogen in water and sediment), autotrophic food, heterotrophic food, and fish. In our model, fish biomass is calculated by the IBM as the sum of the fish mass converted in Kcal (2.17 Kcal.g^-1^ in zebrafish, deduced from the DEB model). Supplementary feed, effect of dissolved oxygen on aerobic process was not integrated. Fish fed only on the heterotrophic compartments, because autotrophic food and cannibalism are supposed to represent a negligible caloric intake for the zebrafish (5.9% of autotrophic food in mean stomach content, Spence et al., 2007). Heterotrophic compartments were modified to integrate the decomposition of the biomass of dead fish (eggs, larvae, juveniles and adults). Finally, over the entire year, the balance between the input and output is supposed null in our food dynamic sub-model. Hence, heterotrophic food and nutriment losses are compensated by inputs during the monsoon period (high food availability during the monsoon months; Spence et al., 2008) which is expressed as a percentage of the initial amount in the pond (*F_in_*).

**Movement** .Wild zebrafish are commonly found in areas of vegetation (Engeszer et al., 2007; Spence et al., 2007). Juvenile fish preferably stay in areas with vegetation which is a defence against predation (Wootton, 1998). Adult zebrafish have shown a preference for breeding grounds characterised by a gravel substrate and vegetation cover as it provides the best habitat for successful reproduction (Spence et al., 2007). Moreover, dominant adult males set up territories in this habitat and defend these sites against other males. In addition, fish length is the main variable determining dominance hierarchies and territoriality in zebrafish (Spence and Smith, 2005; Paull et al., 2008).

Thereby, in the IBM, males, in decreasing length order, move to a free breeding ground patches to set up their territories. If there is no free breeding ground patch, males move randomly into open water patches. Only one female pairs with each male occupying a breeding ground, as pair-wise mating dominates reproductive events (Spence et al., 2007). Therefore, in decreasing length order, mature females (dominant) in reproductive condition (ready to spawn), move to the breeding grounds where males are present and choose the larger one (dominant individuals prioritise mating with dominant individuals of the opposite sex) (Pyron, 2003). The smaller males and females (subordinate) not implied in reproductive event move randomly into open water patches. Male and female dominance hierarchies and territory defence are reset at every time-step.

**Survival**. As very few data are available on zebrafish mortality, survival sub-model was adapted from the one proposed by Hazlerigg et al. (2014). These authors proposed a survival sub-model which includes density-dependent background mortality rates, predation rates, senescent mortality (adults only), and developmental mortality (eggs/larvae only). This survival model is based on a mortality model for freshwater fish developed by Lorenzen (1996) and calibrated on data from laboratory experiments or coming from a natural population. The daily predation mortality probability (*Mp*) was modelled as:

$If L < Lb, M_{p} = \pi_{P}$ Eq. (22)

$If L> Lb, M_{p} = \pi_{a}\times W^{\pi_{b}}$ Eq. (23)

π_a_ is the natural mortality probability at unit weight, $W$ is the weight (mg), π_b_ is an allometric scaling factor and π_p_ is the daily egg predation probability. Hazlerigg et al. (2014) included in their model a density-dependent background mortality probability for all stages. Gerlach (2006) showed that female’s pheromones significantly affect the hatching rate of the other female eggs and that dominant females spawn more viable eggs than subordinate females. Hence, in our model, density-dependent effect was included on the background mortality probability (M_dd_) of the egg/larvae stage only and on hatching rate (detailed in the female fecundity part). Density-dependent background mortality probability (M_dd_) depends of the fish abundance (N):

$M_{dd}=1-\frac{\pi_{c}}{\pi_{d}\times N+1}$ Eq. (24)

π_c_ is the density-independent mortality constant, π_d_ is the density-dependent mortality constant.

A decrease of the survival probability linked to the aging process was also integrated in our model:

$If Age<\pi_{g}, M_{a} = 0$ Eq. (25)

$If Age>\pi_{g}, M_{a} = \pi_{e}\times(Age-\pi_{g})$ Eq. (26)

$\pi_{g}$ is the threshold age above which the mortality increases with age and $\pi_{e}$ is the slope of age and mortality relationship. As no mortality would occur in adult female zebrafish after 21 days of starvation (Drew et al., 2008) and as any other data are available, no explicit modelling of the starvation effect on mortality was modelled.

**Growth**. Growth rate is dependent on water temperature (Eq. S2.1 to S2.4), sex and food and growth in length is predicted by the DEB model . Seasonal modification of growth, which are probably linked to the reproductive status (Wootton, 1998), is ignored in our model. Hence, growth rate is independent of the photoperiod (i.e. reproductive state) but is dependent on the food and temperature conditions. The mass logarithm (mg) / length logarithm (mm) linear relationship fitted on juvenile and adult data (data not shown) was used to predict the mass (parameters W_a_ and W_b_).

**Puberty and sex determination**. Sex ratios across families are wide ranging (from 4.8% to 97.3% reported in Liew et al., 2012) and repeated single pair crossings produced broods of very similar sex ratios, indicating that parental genotypes have a role in the sex ratio of the offspring (Liew et al., 2012). Temperature is unlikely to be the primary signal for zebrafish sex determination, but might exert secondary effects on its sexual development (Liew et al., 2012; Sfakianakis et al., 2012). Hypoxia and growth rate could also impact sex-ratio (Shang et al., 2006; Lawrence et al., 2008). Based on this information, the genetic variation of the sex-ratio and the effect of the water temperature were integrated in our model (two factors with the higher effects). The genetic variation of the sex-ratio was integrated as a stochastic component. Females at each spawning event drawn in a normal distribution fitted on Liew’s data (μ= SR_µ_, σ= SR_σ_, truncated at 0 and 100%) the proportion of males and females in their egg mass ($SR_{g}$). Based on this probability, the individual probability to become a female or a male will be calculated for each juvenile at puberty according to the mean water temperatures experienced from birth to puberty (T_bm_):

$SR_{gt}= SR_{g}\times[ -SR_{a} * T_{bm}+ SR_{b}]$ Eq. (27)

Then, the sex of the juveniles was randomly drawn with a probability $SR_{gt}$ to be a male. The juveniles become males and females at the puberty length (*lp*) which is the length at the first reproductive event (spawn or fertilisation).

**Female inter-spawning interval and fecundity.** Zebrafish spawn during monsoon season, from April to August (Spence et al., 2008) and their reproduction is strongly regulated by photoperiod (Spence et al., 2008). Reproduction period matches with a photoperiod superior to about 12 h, which is a threshold classically observed in other teleost fish (Wootton, 1998). Hence this environmental cue was retained in our model. In lab conditions, when water temperature is reduced to 22.5°C, egg-laying is inhibited (Hisaoka and Firlit, 1962). This second factor delimiting the favourable period for reproduction was also integrated. In our model, we supposed that spawning was induced when a sufficient number of eggs (Rτ; median of all the spawns observed in experiment conducted in our lab: 256 eggs) were produced and if a pair-wise mating with a male was formed.

Egg production rate (*Rm*) of isolated female is dependent on the temperature and food and predicted by the DEB model. Consequently, the lower the temperature is, the longer the inter-spawning interval is.

Olfactory cues play a vital role in zebrafish reproduction. Some steroid glucuronides released into the water by males or by females stimulate the reproductive processes of the fish of the opposite sex but also inhibit the reproductive processes of the fish of same sex (Eaton and Farley, 1974; Gerlach, 2006; Lawrence, 2007). Therefore, based on the data collected by Spence and Smith (2005) a limitation of the female fecundity (*Rm*, maximum eggs production rate) by the female biomass ($D_{female}$ in g/m^3^) was modelled:

$Rm_{t}= Rm \times\left( 1- D_{female} / (H_{0.5}+ D_{female}) \right)$ Eq. (28)

$H_{0.5}$ is the female density decreasing the rate of 50%. As the spawn is induced by an egg number threshold, the female density also impacts the spawning frequency.

Hatching rate is also influenced by density-dependence (Gerlach, 2006). Hatching rate $(H_{d}$) is highly variable among the experiments, from 19.5% to 87% (Markovich et al., 2007; Balasubramani and Pandian, 2008; Uusi-Heikkila et al., 2010). Hence, in our model, hatching rate in a low density population ($H_{max}$) was fixed to the median rate reported in optimal conditions and decreases according to female biomass:

$H_{d}= H_{max}\times(1 - D_{female} / (H_{0.5}+ D_{female}) )$ Eq. (29)

Hisaoka and Firlit (1962) reported that females spawned a large proportion of non-viable eggs 10 days after the last ovulation at 26°C (5 days for a normal cycle at this temperature). Hence, in our model, females excluded from reproduction (i.e., without access to breeding territory) spawn non-viable eggs after the time it takes for two normal cycles (R ≥ 2 × Rτ ) and then restart egg production.

To reduce the computing cost (DEB model calculation for each egg/larva), the age at birth was predicted by the simple function proposed by Kimmel et al. (1995) which is converted in degree.days (H_a_; °C.d^-1^) computed from the threshold H_b_ (°C).

**Inter-individual variability**. Fish length is the main variable determining dominance hierarchies and territoriality in zebrafish (Spence and Smith, 2005; Paull et al., 2008). Dominance in zebrafish has been demonstrated during foraging, where dominant individuals attempt to monopolise a food source (Spence et al., 2008). Moreover, reproductive success is correlated with dominance rank (Gerlach, 2006). However, without inter-individual interaction, in our experiment, females isolated and reared in the same conditions exhibit different egg production rates (Fig. S1.4). This observed variability (coefficient of variation equals to 23%) was mainly due to the differences in female phenotypes. All the different sources of variability of the physiological processes (for instance, variability on growth due both food partitioning between fish and phenotype performance) were integrated in our model through the same parameter, *i.e.* the feeding level. This parameter (*φ_i_*) modulates food consumption, and hence the energy assimilation, the growth rate, the maximal length and the egg productions:

$\varphi_{i} = 1+ Df \times CV_{f} and Df \sim N(0,1)$ Eq. (30)

$CV_{f}$ is the coefficient of variation of the feeding level between the individuals. $Df$ is randomly drawn in *N*(0,1) by each individual at birth for the entire lifespan. The observed variability on egg production in our experiment (mainly related to phenotype variability) was included in the total variability modelled, and informs us on the inferior bound of $CV_{f}$.

Augustine, S., Gagnaire, B., Floriani, M., Adam-Guillermin, C. and Kooijman, S.A.L.M., 2011. Developmental energetics of zebrafish, Danio rerio. pp. 275-283.

Bala, N. and Hasan, M.R., 1999. Seasonal fluctuations in water levels and water quality in Oxbow Lakes in relation in fish yields and social conflict. ICLARM, Conf. Proc., 280 p.

Balasubramani, A. and Pandian, T., 2008. Endosulfan suppresses growth and reproduction in zebrafish. Current Science, 94:10.

Drew, R.E., Rodnick, K.J., Settles, M., Wacyk, J., Churchill, E., Powell, M.S., Hardy, R.W., Murdoch, G.K., Hill, R.A. and Robison, B.D., 2008. Effect of starvation on transcriptomes of brain and liver in adult female. Physiological genomics, 35:283-295.

Eaton, R.C. and Farley, R.D., 1974. Spawning cycle and egg production of zebrafish, Brachydanio rerio, in the laboratory. Copeia, 1:195–204.

Engeszer, R.E., Patterson, L.B., Rao, A.A. and Parichy, D.M., 2007. Zebrafish in the Wild: A Review of Natural History and New Notes from the Field. Zebrafish, 4:21-U126.

Galic, N., Schmolke, A., Forbes, V.E., Baveco, H. and van den Brink, P., 2012. The role of ecological models in linking ecological risk assessment to ecosystem services in agroecosystems. Science of The Total Environment, 415:93-100.

Gerlach, G., 2006. Pheromonal regulation of reproductive success in female zebrafish: female suppression and male enhancement. Animal Behaviour, 72:1119-1124.

Godin, J.-G., 1997. Behavioural Ecology of Teleost Fishes. Oxford University Press, 384 p.

Hazlerigg, C.R.E., Tyler, C.R., Lorenzen, K., Wheeler, J.R. and Thorbek, P., 2014. Population relevance of toxicant mediated changes in sex ratio in fish: An assessment using an individual-based zebrafish (Danio rerio) model. Ecological Modelling, 280:76-88.

Hisaoka, K.K. and Firlit, C.F., 1962. Ovarian cycle and egg production in the zebrafish, *Brachydanio rerio*. Copeia 302-305.

Kimmel, C.B., Ballard, W.W., Kimmel, S.R., Ullmann, B. and Schilling, T.F., 1995. Stages of Embryonic Development of the Zebrafish. Developmental dynamics, 203:255-310.

Lawrence, C., 2007. The husbandry of zebrafish (*Danio rerio*): A review. Aquaculture, 269:1-20.

Lawrence, C., Ebersole, J.P. and Kesseli, R.V., 2008. Rapid growth and out-crossing promote female development in zebrafish (Danio rerio). pp. 239-246.

Li, L. and Yakupitiyage, A., 2003. A model for food nutrient dynamics of semi-intensive pond fish culture. Aquacultural Engineering, 27:9-38.

Liew, W.C., Bartfai, R., Lim, Z., Sreenivasan, R., Siegfried, K.R. and Orban, L., 2012. Polygenic Sex Determination System in Zebrafish. PLoS ONE, 7:e34397.

Lorenzen, K., 1996. The relationship between body weight and natural mortality in juvenile and adult fish: a comparison of natural ecosystems and aquaculture. Journal of Fish Biology, 49:627-642.

Markovich, M.L., Rizzuto, N.V. and Brown, P.B., 2007. Diet Affects Spawning in Zebrafish. Zebrafish, 4:67-74.

Paull, G.C., Van Look, K.J.W., Santos, E.M., Filby, A.L., Gray, D.M., Nash, J.P. and Tyler, C.R., 2008. Variability in measures of reproductive success in laboratory-kept colonies of zebrafish and implications for studies addressing population-level effects of environmental chemicals. Aquatic Toxicology, 87:115-126.

Pyron, M., 2003. Female preferences and male–male interactions in zebrafish (Danio rerio). Canadian Journal of Zoology, 81:122-125.

Sfakianakis, D.G., Leris, I., Mylonas, C.C. and Kentouri, M., 2012. Temperature during early life determines sex in zebrafish, Danio rerio (Hamilton, 1822). Journal of Biological Research-Thessaloniki, 17:68-73.

Shang, E.H., Yu, R.M. and Wu, R.S., 2006. Hypoxia affects sex differentiation and development, leading to a male-dominated. Environ Sci Technol, 40:3118-3122.

Spence, R., Fatema, M.K., Ellis, S., Ahmed, Z.F. and Smith, C., 2007. Diet, growth and recruitment of wild zebrafish in Bangladesh. Journal of Fish Biology, 71:304-309.

Spence, R., Gerlach, G., Lawrence, C. and Smith, C., 2008. The behaviour and ecology of the zebrafish, Danio rerio. Biol Rev Camb Philos Soc, England, pp. 13-34.

Spence, R. and Smith, C., 2005. Male territoriality mediates density and sex ratio effects on oviposition in the zebrafish, Danio rerio. Animal Behaviour, 69:1317-1323.

Ulloa, P.E., Iturra, P., Neira, R. and Araneda, C., 2012. Zebrafish as a model organism for nutrition and growth: towards comparative studies of nutritional genomics applied to aquacultured fishes. Reviews in Fish Biology and Fisheries, 21:649-666.

Uusi-Heikkila, S., Wolter, C., Meinelt, T. and Arlinghaus, R., 2010. Size-dependent reproductive success of wild zebrafish *Danio rerio* in the laboratory. Journal of Fish Biology, 77:552-569.

Wootton, R.J., 1998. Ecology of Teleost Fishes. Kluwer Academic Publishers, Dordecht, The Nethrelands. , 386 p.
