# Supplementary material for: An Individual-Based Model of Zebrafish Population Dynamics Accounting for Energy Dynamics
Source: PLoS One. 2015 May 4;10(5):e0125841. doi: 10.1371/journal.pone.0125841 (PMC4418570; doi:10.1371/journal.pone.0125841)
Supplement: S4 Text — (DOCX) [file pone.0125841.s006.docx]

Appendix S4. Netlogo code of the zebrafish ibm

- Model was developed using NetLogo 5.0.4 (March 19, 2013).
- Content of the text file ("InputDataFromMay.txt"), which includes the temperatures and photoperiods, was provided below the netlogo code.

;; Rémy Beaudouin and Benoit Goussen

;; Zebrafish agent-based model based on a DEB model

;_____________________________________________________________________________________________________________________________

; Agents

;_____________________________________________________________________________________________________________________________

breed [ Juveniles Juvenile ]

breed [ Males Male ]

breed [ Females Female ]

breed [ EggMasses EggMass]

turtles-own [Generation Variability Age SR M.R M.M M.Age S.rate Test]

Juveniles-own [Sexe L W R NRJ dL dR dNRJ food.i Ec.i t.puberty SR.temperature ]

Males-own [Sexe L W R NRJ dL dR dNRJ food.i Ec.i territory Engaged]

Females-own [Sexe L W R NRJ dL dR dNRJ food.i Ec.i Rm.i ]

EggMasses-own [Neggs Sr.L DD.i H.rate ]

;_____________________________________________________________________________________________________________________________

; Cellule

;_____________________________________________________________________________________________________________________________

patches-own [Habitat Male.Tenant ]

;_____________________________________________________________________________________________________________________________

; Variables and parameter globals

;_____________________________________________________________________________________________________________________________

globals[

;------------------------------------ model variables ------------------------------------

Player W.Tot W.female fish.D Female.D End-Experiment pdt

Food f_N_P f_I f_T f_h FB FBdead

TIP TIN TNS TPS Neva Nfix Ec HFc Af Hf

Temperature Jour.Temp TempW Photoperiod JourPhoto Photo SSQ

;------------------------------------ model parametres ------------------------------------

Tc Ta Tr Wv Wd Nb.J Nb.F Nb.M Np.b Np.v FoodInput ;; Environment

I_r a_I b_I T_opta k_T1 K_T2 Kn Kpr Kps s ;; Food (States Parameters)

r_max K_r K_ml Kap Kan K_s K_d Khn Khp ;; Food (States Parameters)

G2Kcal h_n h_p Ksn Knl ;; Food (States Parameters)

Kappa v PAm PM Km Eg alpha lf sdi PM.t v.t PAm.t ;; energy

L.p lp Rm P.t H.max H.0.5 R.t SR.mu SR.sd SR.a SR.b s.dmax T.t ;; Puberty and Reproduction

M.a M.b M.c M.d M.p M.e M.g ;; Survival

Aff Zota lb L.b Linf Em g Fl.m aW bW a.h b.h ;; Growth

;------------------------------------ model outputs ------------------------------------

N.tot N.tot.adult N.Fond ; Abundance

F.M F.F F.J ; Frequency (Males, Females, Juveniles)

L.J L.M L.F ; Mean length

CV.J CV.M CV.F ; CV length

L.FreqM L.FreqF L.FreqJuv L.FreqT ; Length frequency distribution

]

;_____________________________________________________________________________________________________________________________

; Model initialization

;_____________________________________________________________________________________________________________________________

to SETUP

clear-all

set-default-shape Juveniles "fish"

set-default-shape Males "fish"

set-default-shape Females "fish"

set-default-shape EggMasses "dot"

Inputs

Model-parameters

Setup-patches

Setup-environment

Setup-individual

reset-ticks

end

;_____________________________________________________________________________________________________________________________

; Simulation

;_____________________________________________________________________________________________________________________________

to GO

;; Environment variables

Update-environment

Update-food

;; Agent behaviour

Move

Survive

DEBmodel

Hatching

Puberty

Spawn

Aging

;; Time simulation

tick

CalcOutputs

if ticks = End-Experiment [stop]

end

;_____________________________________________________________________________________________________________________________

; Simulation initialisation

;_____________________________________________________________________________________________________________________________

to Setup-environment

set Photoperiod select-list-T Jour.Temp Photo 0

set Temperature select-list-T Jour.Temp TempW 0

set Tc exp( Ta / Tr - Ta / (Temperature + 273 ) )

set Af 2.5 * Wv ; Kcal/pond

set Hf 18.4 * Wv ; Kcal/pond

set TIN 0.97 * Wv ; total dissolved inorganic nitrogen (g / pond)

set TIP 0.02 * Wv ; total dissolved inorganic phosphorus (g / pond)

set TNS TIN ; total nitrogen in sediment (g / pond)

set TPS TIP ; total phosporus in sediment (g / pond)

end

to Setup-patches

ask patches[

set Habitat "OpenWater"

set pcolor 96

set Male.Tenant 0 - 1 ]

ask n-of Np.b patches with [ Habitat = "OpenWater" ][

set Habitat "BreedingGrounds"

set pcolor 94 ]

ask n-of Np.v patches with [ Habitat = "OpenWater" ] [

set Habitat "Vegetation"

set pcolor 95 ]

end

to Setup-individual

create-Juveniles Nb.J [

Initialisation-Juveniles

set SR normal-seuil SR.mu SR.sd 0 100

set Generation 0

set L ( random-float (L.p - L.b ) ) + L.b

set Age 287 * L / Linf ]

create-Males Nb.M [

set L random-float (Linf - L.p ) + L.p

set W exp( aW * ln L - bW )

set Age 287 * L / Linf

set Variability (random-normal 0 1 )

set Generation 0

Initialisation-Males

setxy random-xcor random-ycor]

create-females Nb.F [

set L random-float (Linf - L.p ) + L.p

set W exp( aW * ln L - bW )

set Age 287 * L / Linf

set Variability (random-normal 0 1 )

set Generation 0

Initialisation-Females ]

;; Turtles juveniles, females, males

set Player turtles with [ breed != eggmasses]

;; biomass

set N.tot count Player ; Total number of individuals (Juveniles + Males + Females)

set W.Tot (sum [W] of Player ) / 1000 ; Fish biomass (g)

set W.female (sum [W] of Females) / 1000 ; Females biomass (g)

set fish.D (N.tot / Wv ) ; Fish density ( g / m3 )

set Female.D (W.female / Wv ) ; Females density ( g / m3 )

set FB W.Tot * G2Kcal ; Fish biomass in Kcal/pond

;; Fish food consumption (HFCi / HFC )

set f_h ( 1 - exp( - s * ( Hf / (FB + 1E-5) )^ 2.2 )) ; food aviability, if FB= 0 --> 1E-5

ask Player [ set Ec.i PAm * 1 * ( L * zota ) ^ 2 * (1 / 4186.8) ] ; nrj feeds ad-libitum in Kcal

set Ec ( sum [Ec.i] of player)

set HFc Ec * f_h ; global nrj feeds in Kcal

end

;_____________________________________________________________________________________________________________________________

; Non fish elements updates (Food, tempertaure, photoperiod)

;_____________________________________________________________________________________________________________________________

to Update-environment

set Photoperiod select-list-T Jour.Temp Photo ticks

set Temperature select-list-T Jour.Temp TempW ticks

;; correct all parameters depending on time to temperature

set Tc exp( Ta / Tr - Ta / (Temperature + 273 ) )

set v.t v * Tc ; Energy conductance (mm /d)

set PAm.t PAm * Tc ; Maximum area specific assimilation rate (J/ d / mm^2)

set PM.t PM * Tc ; Volume somatic maintenance costs (J/d/mm^3)

set Km PM.t / Eg ; Somatic maintenance rate

;; Turtles juveniles, females, males

set Player turtles with [ breed != eggmasses]

;; biommase

set N.tot count Player ; Total number of individuals (Juveniles + Males + Females)

set W.Tot (sum [W] of Player ) / 1000 ; Fish biomass (g)

set W.female (sum [W] of Females) / 1000 ; Females biomass (g)

set fish.D (N.tot / Wv ) ; Fish density ( g / m3 )

set Female.D (W.female / Wv ) ; Females density ( g / m3 )

set FB (W.Tot * G2Kcal ) ; Fish biomass in Kcal/pond

ask patches[ set Male.Tenant (- 1) ]

end

to Update-food

;; Temperature function

ifelse( Temperature <= T_opta )

[set f_T exp( - k_T1 * ( Temperature - T_opta )^ 2 ) ]

[ set f_T exp( - k_T2 * (T_opta - Temperature )^ 2 ) ]

;; solar radiation function

set f_I ( I_r * exp( -(a_I * AF + b_I * Hf) ) ) / I_r

;; heterotrophic food consumption and availability for fish (dimensionless)

set f_h ( 1 - exp( - s * ( Hf / (FB + 1E-5) )^ 2.2 )) ; Food aviability, if FB= 0 --> 1E-5

set Ec sum [Ec.i] of Player ; Global fish energy demands (Kcal)

set HFc Ec * f_h ; Global fish energy consumed (Kcal)

;; Variables computation

let INC TIN / Wv ;concentration in mg/L

let IPC TIP / Wv ;concentration in mg/L

let N.conc INC / ( INC + h_n ) ;total inorganic nitrogen mg/L

let P.conc IPC / ( IPC + h_p ) ;total inorganic phodporus mg/L

ifelse (N.conc < P.conc ) [ set f_N_P N.conc ] [ set f_N_P P.conc ]

let AFR K_r * AF ;autotrpohic food loss due to phytoplankton loss

let AFG r_max * f_N_P * f_I * f_T * AF ;autotrophic food growth due to phytoplankton growth (kcal day-1 pond-1)

let AFM K_ml * AF ;A food to H food pool due to phytop mort. and harvest by secondary producers

let HFD K_d * HF ;heterotrophic food loss rate (kcal day-1 pond-1) due to decomposition

let HFS K_s * HF ;heterotrophic food loss rate (kcal day-1 pond-1) due to sedimentation

;; Nitrogen dynamics

set Neva TIN * Knl

set Nfix Kn * AF * exp( - Kn * ( INC ^ 2 ) )

let Naf AFG * Kan - Nfix

let dTIN AFR * Kan + HFD * Khn + TNS * Ksn - Naf - Neva

let dTNS HFS * Khn - TNS * Ksn

set TIN Positif ( TIN + dTIN ) 0

set TNS Positif ( TNS + dTNS ) 0

;; Phosphorus dynamic

let dTIP AFR * Kap + HFD * Khp + (TPS * Kpr / Wd ) - AFG * Kap - (TIP * Kps / Wd)

let dTPS HFS * Khp + (TIP * Kps / Wd) - (TPS * Kpr / Wd)

set TIP Positif ( dTIP + TIP ) 0

set TPS Positif ( dTPS + TPS ) 0

;; Autotrophic food dynamics

let dAf (AFG - AFR - AFM)

set Af Positif ( Af + dAf ) 1E-6

;; Heterotrophic food dynamics

let dHf FBdead + AFM - HFS - HFD - HFC

set Hf Positif ( Hf + dHf ) 1E-6

;; nutriment input during monsoon period

if( Photoperiod > P.t )[

set TIN TIN + FoodInput * ( 0.97 * Wv )

set TIP TIP + FoodInput * ( 0.01 * Wv )

set Hf Hf + FoodInput * ( 18.4 * Wv )

]

set FBdead 0 ; initialisation time step variable

end

;_____________________________________________________________________________________________________________________________

; fish updates

;_____________________________________________________________________________________________________________________________

; Initialisation des agents

;-------------------------------------

to Initialisation-Juveniles

Set L L.b

set Sexe"F"

set NRJ 1

setxy random-xcor random-ycor

set color grey

set size 0.5

set W exp( aW * ln L - bW )

set Variability (random-normal 0 1 )

end

to Initialisation-Males

set color 23

set size 1

set shape "fish"

setxy random-xcor random-ycor

set NRJ random-float 1

set sexe "M"

end

to Initialisation-Females

set color 43

set size 1.1

set shape "fish"

setxy random-xcor random-ycor

set NRJ random-float 1

set R random-float R.t

set sexe "F"

end

; Movement

;-------------------------------------

to Move ;; turtle procedure

ask Juveniles [ let p.me one-of patches with [Habitat = "Vegetation" ] move-to p.me ]

ask Females [ rt random-float 360 fd random 2 ]

let M.size sort-by [[L] of ?1 > [L] of ?2] Males

foreach M.size [ ask ? [

let p.me one-of patches with [Habitat = "BreedingGrounds" and Male.Tenant = -1 ]

ifelse( p.me != nobody )[ ; First case free breeding ground patches

move-to p.me

set territory 1

ask p.me [ set Male.Tenant [who] of myself]

][ ; Second case No free breeding ground patches.

lt random-float 360 fd random 2 ; if no territory = move random

] ]]

end

; Aging

;-------------------------------------

to Aging

ask turtles [ set Age Age + 1 ]

end

; DEBmodel

;-------------------------------------

to DEBmodel

ask Player [

;; individual fish heterotrophic food consumption (HFCi / HFC )

set food.i bound ( f_h + Variability * ( sdi * f_h) ) 0 1

set Ec.i PAm.t * 1 * ( L * zota ) ^ 2 * (1 / 4186.8) ; nrj feeds ad-libitum in Kcal

;;feeding motivation change after puberty

if (breed = males)[ set food.i (Fl.m * food.i) ]

;; Eggs porduction rate related to female density

if( breed = females )[ set Rm.i Rm * (1 - female.D /(H.0.5 + female.D) ) ]

;; intialisation

let l.dyn ( L / Linf ) ; scaled length

let r.dyn R

let e.dyn NRJ

let d.e 0

let d.r 0

let d.l 0

let F.lim 0

foreach (n-values (1 / pdt) [?]) [

;; Stress alimentation

set F.lim ( 1 - ( ( 1 - ( 1 / ( 1 + ( lf ^ 3 / l.dyn ^ 3 ))) ) * alpha ) )

;; Energie

set d.e ( ( ( Km * g ) / l.dyn ) * ( F.lim * Food.i - e.dyn ) * pdt )

;; Growth

let rB ( Km * g ) / ( 3 * ( e.dyn + g ) )

set d.l ( rB * ( e.dyn - l.dyn ) * pdt )

if ( d.l < 0 ) [set d.l 0]

;; Reproduction model

if( breed = males )[ set d.r (( 1 / ( 1 - lp ^ 3) * (( (g + l.dyn ) / (g + e.dyn) ) * e.dyn * l.dyn ^ 2 - lp ^ 3 )) * pdt )]

if( breed = females)[ set d.r (( Rm.i / ( 1 - lp ^ 3) * (( (g + l.dyn ) / (g + e.dyn) ) * e.dyn * l.dyn ^ 2 - lp ^ 3 )) * pdt )]

if( ( l.dyn < lp ) or ( dr < 0 ) )[ set d.r 0 ] ;; breed = juveniles

;; Environmental control on reproduction

if( ( Photoperiod < P.t) or (Temperature < T.t) )[ set d.r 0 ]

;; iteration

set e.dyn e.dyn + d.e

set l.dyn l.dyn + d.l

set r.dyn r.dyn + d.r

]

set dNRJ e.dyn - NRJ ; energy dynamic

set NRJ e.dyn ; Energy at t+1

set dL l.dyn - (L / Linf)

set L l.dyn * Linf ; Length at t+1

set dR r.dyn - R

set R r.dyn ; Number of eggs at t + 1

set W exp( aW * ln L - bW ) ; mass (mg)

]

end

;; Death

;-------------------------------------

to Survive

ask EggMasses [

set M.R ( 1 - ( M.c / ( 1 + ( M.d * N.tot ) ) ) ) ; Density-dependent background mortality probability

set M.M M.P ; Daily predation mortality

set S.rate 1 - ( M.M + M.R ) ; Daily survival rate

let tmp ( binomial-drawn Neggs S.rate)

set FBdead FBdead + ( 0.16 / 1000 * G2Kcal * (Neggs - tmp) ) ; 0.16 mg larvae mass

set Neggs tmp ] ; Number of surviving eggs

ask Player [

set M.M ( M.a * W ^ M.b ) ; Daily predation mortality

ifelse( Age < M.g)[ set M.Age 0 ][ set M.Age M.e * (Age - M.g) ] ; Ageing mortality

set S.rate ( 1 - ( M.M + M.Age)) ; Global daily survival rate

if( S.rate * 100 ) <= (random-float 100) [

set FBdead FBdead + ( W / 1000 * G2Kcal )

die ]

]

end

;; Puberty

;-------------------------------------

to Puberty

ask Juveniles [

set t.puberty t.puberty + 1

set SR.temperature SR.temperature + Temperature ; cumulated temperature until puberty

]

ask Juveniles with [L >= L.p][

set SR.temperature SR.temperature / t.puberty

set SR SR + ( SR.a *( SR.temperature - SR.b ))

set SR bound SR 0 100 ; Apply a boundary to SR

if SR >= (random-float 100) [ set sexe "M" ] ; SR is male frequency

if(sexe = "M")[ set breed Males Initialisation-Males ]

if(sexe = "F")[ set breed females Initialisation-females ] ]

end

;; spawning

;-------------------------------------

to Spawn

let F.size sort-by [[L] of ?1 > [L] of ?2] Females with [R > R.t ]

foreach F.size [ ask ?[

let Partner max-one-of males with [Territory = 1 and Engaged = 0 ] [L]

ifelse ( Partner != nobody )[

; move-to Partner

ask Partner[ set Engaged 1 ]

hatch-EggMasses 1 [

set H.rate H.max * ( 1 - female.D / ( H.0.5 + female.D ) )

set Generation ([Generation] of myself + 1)

set Neggs [R] of myself

set color yellow

set SR normal-seuil SR.mu SR.sd 0 100

set Age 0

set DD.i 0

setxy ([xcor] of Partner) ([ycor] of Partner)

set size 0.5 ]

set R 0

][

if (R > ( 2 * R.t ) ) [ set R 0 ] ; eggs are lost

] ] ]

ask males [ set Territory 0 set Engaged 0] ; initialisation reproduction

end

;; Hatching

;-------------------------------------

to Hatching

ask EggMasses [

set DD.i DD.i + ( Temperature - b.h )

if ( a.h <= DD.i ) [

hatch-Juveniles NEggs * H.rate[

Initialisation-Juveniles

set SR ([SR] of myself)

set Generation ([Generation] of myself ) ]

die ]

]

end

;_____________________________________________________________________________________________________________________________

; OUTPUTS : Les sorties du modele en fin de simulation

;_____________________________________________________________________________________________________________________________

to CalcOutputs

set N.tot.adult count Males + count Females ; Total number of adults (Males + Females)

ifelse(N.tot > 0)[set F.m count Males / N.tot * 100 ][set F.M 0] ; Males frequency

ifelse(N.tot > 0)[set F.F count Females / N.tot * 100 ][set F.F 0] ; Females frequency

ifelse(N.tot > 0)[set F.J count Juveniles / N.tot * 100 ][set F.J 0] ; Juveniles frequency

set N.Fond count turtles with [Generation = 0] ; Number of fish introduced at the onset of experiment

ifelse ( count Juveniles = 0)[set L.J 0 ][set L.J mean [L] of Juveniles] ; juveniles mean length

ifelse ( count Males = 0 )[set L.M 0 ][set L.M mean [L] of Males ] ; males mean length

ifelse ( count Females = 0 )[set L.F 0 ][set L.F mean [L] of Females ] ; females mean length

ifelse (count Juveniles <= 1)[set CV.J 0][set CV.J 100 *(standard-deviation [L] of Juveniles) / mean [L] of Juveniles];juv length CV

ifelse (count Males <= 1 )[set CV.M 0 ][set CV.M 100 * (standard-deviation [L] of Males ) / mean [L] of Males ] ;males length CV

ifelse (count Females <= 1 )[set CV.F 0 ][set CV.F 100 * (standard-deviation [L] of Females) / mean [L] of Females ] ;females length CV

set L.FreqF Histo 80 Females 1 ; Call function Histo[Bsup Stage Step]

set L.FreqM Histo 80 Males 1 ; Call function Histo[Bsup Stage Step]

set L.FreqJuv Histo 26 Juveniles 1 ; Call function Histo[Bsup Stage Step]

set L.FreqT Histo 80 Player 1 ; Call function Histo[Bsup Stage Step]

end

;_____________________________________________________________________________________________________________________________

; INPUTS : Les entrees du modele

;_____________________________________________________________________________________________________________________________

to Inputs

;; water temperature and photoperiod

;;----------------

file-open "InputDataFromMay.txt"

set Jour.Temp [] set TempW [] set Photo []

while [ not file-at-end? ] [

set Jour.Temp sentence Jour.Temp (list file-read)

set TempW sentence TempW (list file-read)

set Photo sentence Photo (list file-read)]

file-close

end

;_____________________________________________________________________________________________________________________________

; Modes parameters

;_____________________________________________________________________________________________________________________________

to Model-parameters

;; Model inputs

; --------------

set pdt 0.1 ; realtive time step of DEB model (pdt = DEB-pdt/IBM-pdt = 2h/24h = 0.083)

;# "initial-number-males", "initial-number-females", "initial-number-juveniles" and "experiment-duration" are defined on the interface

;; set End-Experiment experiment-duration ; (days) experience duration (selected on interface (default 800))

;; set Nb.F initial-number-females ; initial nb of males (selected on interface (default 10))

;; set Nb.M initial-number-males ; initial nb of females (selected on interface (default 10))

;; set Nb.J initial-number-juveniles ; initial nb of juveniles (selected on interface (default 0))

;# To work whitout the interface

set End-Experiment 1111 ; (days) experience duration (selected on interface (default 800))

set Nb.F 35 ; initial nb of males (selected on interface (default 10))

set Nb.M 35 ; initial nb of females (selected on interface (default 10))

set Nb.J 300 ; initial nb of juveniles (selected on interface (default 0))

;; Environment

; --------------

set Wv 18 ; water volume (m3) L 6m x l 6m x d 0.5m (selected on interface (default 1))

set Wd 0.5 ; water depth (m)

set Np.b 207 ; 23% breeding grounds

set Np.v 207 ; 23% vegetation cover

;; Food

; --------------

set FoodInput 0.0142

set s 21.08 ; proportionality coefficient of food nutrient quantity to fish biomass (dimensionless)

; nutriment dynamic

set h_n 0.2 ; half saturation Nitrogen (mgN/L)

set h_p 0.02 ; half saturation Phosporus (mgP/L)

set Kn 0.01 ; N-fixation coefficient of phytoplankton

set Kan 0.0224 ; N content of phytoplankton

set Khn 0.0192 ; N content of heterotrophic components

set Ksn 0.003 ; release coefficient of nitrogen in sediment

set Knl 0.17 ; coefficient of inorganic nitrogen loss to air

set Kap 0.001 ; P content of phytoplankton

set Khp 0.001 ; P content of heterotrophic components

set Kpr 0.0006 ; release coefficient of phosphorus in sediment (m.day-1)

set Kps 0.28 ; coefficient of inorganic phosphorus sedimentation to sediment (m.day-1)

; Heterotrophic food dynamics

set K_s 0.14 ; coefficient of heterotrophic food sedimentation (day-1),

set K_d 0.12 ; oefficient of heterotrophic food decomposition (day-1),

; Autotrohic food cpt

set r_max 1.6 ; maximum growth coefficient for phytoplankton growth (day-1)

set K_r 0.1 ; coefficient of phytoplankton respiration (day^-1),

set K_ml 0.6 ; coefficient of A food entering H food pool (phytoplankton mortality and harvest; day^-1)

; limiting functions of solar radiation

set I_r 6.547 ; reference solar radiation 10^6 cal/ m^2/day

set a_I 0.000017 ; li et al. 2003 ; pond/Kcal

set b_I 0.000015 ; li et al. 2003 ; pond/Kcal

; Limiting functions of temperature

set T_opta 30 ; optimal temperature for phytoplankton growth (°C)

set k_T1 0.004 ; effects of temperature below Topta on growth (°C-2).

set K_T2 0.008 ; effects of temperature above Topta on growth (°C-2).

;; DEB parameter

;---------------------------

set PAm 4.72 ; Maximum area specific assimilation rate (J/ d / mm^2) # <- 2.463

set v 0.60 ; Energy conductance (mm /d)

set Kappa 0.70 ; fraction of energy to growth/somatic

set sdi 0.235 ; variability of the energy acquisition

set lb 0.084 ; Scaled length at birth

set lf 0.156 ; scaled length at half maximal assimilation

set lp 0.58 ; scaled length at puberty (-)

set alpha 0.84 ; fraction inaccessible

set PM 0.44 ; Volume somatic maintenance costs (J/d/mm^3)

set Eg 2.35 ; Cost of synthesis of a unit of structure (J/mm^3)

set Ta 3000 ; Arrhenius temperature (k)

set Tr 293 ; Reference temperature (k)

set Rm 406 ; maximum number of egg/ day EatoFarl1974b T= 25.5 + 273 K

set Zota 0.20 ; Shape coefficient for adults V = zota * L ^3

;; Puberty and Reproduction

;---------------------------

set P.t 12 / 24 ; Photoperiod threshold to the onset of the reproductive activity

set T.t 22.5 ; Temperature threshold to the on/offset of the reproduction

set H.0.5 24 ; Fish density inducing 50 % reduction fecundity

set H.max 0.89 ; Hatching rate optimal

set R.t 263 ; Number of eggs neccesary to spawn

set SR.mu 50 ; sex-ratio genetic variability

set SR.sd 23.1 ; sex-ratio genetic variability

set SR.a (- 0.0496) ; slope sex-ratio genetic f(water temperature)

set SR.b 27.9 ; sex-ratio genetic °C

set a.h 60.9 ; age at first feeding expressed in degree.day

set b.h 10.3 ; Temperature threshold degree.day age at first feeding

;; Survival

;---------------------------

set M.a 0.0292 ; natural mortality probability at unit weight

set M.b (- 0.382) ; an allometric scaling factor

set M.c 0.9576 ; density-independent mortality constant

set M.d 0.0089 ; slope density-dependent mortality constant

set M.p 0.025 ; the daily egg predation probability

set M.g 550 ; Age threshold of mortality due to aging

set M.e 2.839e-06 ; Ageing mortality

;; Growth

;----------------------------

set Fl.m 0.91 ; Male appetite modified by male puberty

set aW 3.205 ; allometric relation L/W

set bW 5.1928 ; allometric relation L/W

;; Parameters functions of primary parameters

;---------------------------

set Em PAm / v ; maximum reserve density (J/mm^3)

set g Eg / (Kappa * Em) ; energy investment ratio

set Linf (v / ( ( PM / Eg ) * g ) ) * 1 / Zota ; Maximum physical length (mm)

set L.p lp * Linf ; (mm) length at puberty

set L.b lb * Linf ; length at birth (mm)

set G2Kcal Em * 1000 / 4186 ; zebrafish Kcal/g ; 1 Kcal = 4186 J ; d = 1 g/cm3 lindsey et al. 2010

end

;_____________________________________________________________________________________________________________________________

; Reporter

;_____________________________________________________________________________________________________________________________

to-report L.Moy [Sx Co]

ifelse count turtles with [breed = Co and sexe = Sx] > 0

[ report mean [L] of turtles with [breed = Co and sexe = Sx] ]

[ report 0 ]

end

to-report R-Ad-F

ifelse count females > 0

[ report mean [R] of Females ]

[ report 0 ]

end

to-report select-list-T [ListC ListD val2]

let valtmp val2

if( ( val2 / 365 ) > 1 ) [ set valtmp ( val2 - ( 365 * floor (val2 / 365) ) ) ]

let A (position valtmp ListC)

report item A ListD

end

to-report normal-seuil [Moy Sd Tha Thb]

let tirage random-normal(Moy) (Sd)

while [(tirage < Tha) or (tirage > Thb )][ set tirage random-normal(Moy) (Sd) ]

report tirage

end

to-report Histo [UppB Stage Step] ; Histogram of individual length of values from 0 to Bsup for selected stade (Juvenile, Male, Female) with a step of "step" mm

let temp []

let Inter n-values UppB [?]

foreach Inter [set temp lput (frequency (?) ([L] of Stage) (Step) ) temp ]

report temp

end

to-report HistoLowerLT [LowerB UppB Stage Step] ; Histogram total lenght Ls * 1.25 = LT

let temp []

let Inter (n-values ( UppB - LowerB ) [?] )

foreach Inter [set temp lput (frequency (? + LowerB ) (([L * 1.25] of Stage) ) (Step) ) temp ]

report temp

end

to-report frequency [val thelist Step] ; Frequency of values between val and val+Step

report length filter [(? >= val) and (? < (val + Step))] thelist

end

to-report bound [Nbr LowB UppB] ; Apply boundary to a number

let tmp Nbr

if( Nbr > UppB ) [ set tmp UppB ]

if( Nbr < LowB ) [ set tmp LowB ]

report tmp

end

to-report Positif [Nbr LowBr] ; Apply boundary to a number

let tmp Nbr

if( Nbr < LowBr ) [ set tmp LowBr ]

report tmp

end

to-report binomial-drawn [n proba.binom ]

let res.binom 0

let l.n n-values n [?]

foreach l.n [ if random-float 1 < proba.binom [ set res.binom res.binom + 1] ]

report res.binom

end

Content of the "InputDataFromMay.txt" file

0 29.74666667 0.541053241

1 29.79333333 0.54181713

2 29.84 0.542569444

3 29.88666667 0.543310185

4 29.93333333 0.544050926

5 29.98 0.544768519

6 30.02666667 0.545486111

7 30.07333333 0.54619213

8 30.12 0.546898148

9 30.16666667 0.547581019

10 30.21333333 0.548263889

11 30.26 0.548923611

12 30.30666667 0.549583333

13 30.35333333 0.550219907

14 30.4 0.550856481

15 30.38387097 0.551481481

16 30.36774194 0.552083333

17 30.3516129 0.552673611

18 30.33548387 0.553263889

19 30.31935484 0.553831019

20 30.30322581 0.554386574

21 30.28709677 0.554918981

22 30.27096774 0.555451389

23 30.25483871 0.555960648

24 30.23870968 0.556458333

25 30.22258065 0.55693287

26 30.20645161 0.557407407

27 30.19032258 0.557858796

28 30.17419355 0.558287037

29 30.15806452 0.558703704

30 30.14193548 0.559108796

31 30.12580645 0.559490741

32 30.10967742 0.559861111

33 30.09354839 0.560208333

34 30.07741935 0.560543981

35 30.06129032 0.560856481

36 30.04516129 0.561157407

37 30.02903226 0.561435185

38 30.01290323 0.561689815

39 29.99677419 0.56193287

40 29.98064516 0.562152778

41 29.96451613 0.562361111

42 29.9483871 0.562546296

43 29.93225806 0.562708333

44 29.91612903 0.562858796

45 29.9 0.562986111

46 29.89166667 0.563090278

47 29.88333333 0.56318287

48 29.875 0.563252315

49 29.86666667 0.563298611

50 29.85833333 0.563321759

51 29.85 0.563333333

52 29.84166667 0.563321759

53 29.83333333 0.563287037

54 29.825 0.563240741

55 29.81666667 0.563171296

56 29.80833333 0.563078704

57 29.8 0.562974537

58 29.79166667 0.562847222

59 29.78333333 0.562696759

60 29.775 0.562534722

61 29.76666667 0.562337963

62 29.75833333 0.562141204

63 29.75 0.561909722

64 29.74166667 0.561666667

65 29.73333333 0.561412037

66 29.725 0.561134259

67 29.71666667 0.560833333

68 29.70833333 0.560520833

69 29.7 0.560185185

70 29.69166667 0.559837963

71 29.68333333 0.559467593

72 29.675 0.559085648

73 29.66666667 0.55869213

74 29.65833333 0.558275463

75 29.65 0.557835648

76 29.64516129 0.557384259

77 29.64032258 0.556921296

78 29.63548387 0.556446759

79 29.63064516 0.555949074

80 29.62580645 0.555439815

81 29.62096774 0.554918981

82 29.61612903 0.554386574

83 29.61129032 0.553831019

84 29.60645161 0.553275463

85 29.6016129 0.552696759

86 29.59677419 0.552106481

87 29.59193548 0.55150463

88 29.58709677 0.550891204

89 29.58225806 0.550266204

90 29.57741935 0.54962963

91 29.57258065 0.548981481

92 29.56774194 0.548321759

93 29.56290323 0.547662037

94 29.55806452 0.546979167

95 29.55322581 0.546284722

96 29.5483871 0.545590278

97 29.54354839 0.544884259

98 29.53870968 0.544166667

99 29.53387097 0.5434375

100 29.52903226 0.542696759

101 29.52419355 0.541956019

102 29.51935484 0.541203704

103 29.51451613 0.540451389

104 29.50967742 0.539675926

105 29.50483871 0.538912037

106 29.5 0.538125

107 29.50322581 0.537337963

108 29.50645161 0.536550926

109 29.50967742 0.535752315

110 29.51290323 0.53494213

111 29.51612903 0.534131944

112 29.51935484 0.533321759

113 29.52258065 0.5325

114 29.52580645 0.531666667

115 29.52903226 0.530844907

116 29.53225806 0.53

117 29.53548387 0.529166667

118 29.53870968 0.528321759

119 29.54193548 0.527476852

120 29.54516129 0.52662037

121 29.5483871 0.525763889

122 29.5516129 0.524907407

123 29.55483871 0.524039352

124 29.55806452 0.523171296

125 29.56129032 0.522303241

126 29.56451613 0.521435185

127 29.56774194 0.520555556

128 29.57096774 0.519675926

129 29.57419355 0.518796296

130 29.57741935 0.517916667

131 29.58064516 0.517037037

132 29.58387097 0.516145833

133 29.58709677 0.51525463

134 29.59032258 0.514375

135 29.59354839 0.513483796

136 29.59677419 0.512592593

137 29.6 0.511689815

138 29.57833333 0.510798611

139 29.55666667 0.509907407

140 29.535 0.509016204

141 29.51333333 0.508113426

142 29.49166667 0.507222222

143 29.47 0.506319444

144 29.44833333 0.505416667

145 29.42666667 0.504525463

146 29.405 0.503622685

147 29.38333333 0.502719907

148 29.36166667 0.501828704

149 29.34 0.500925926

150 29.31833333 0.500034722

151 29.29666667 0.499143519

152 29.275 0.498240741

153 29.25333333 0.497349537

154 29.23166667 0.496458333

155 29.21 0.495555556

156 29.18833333 0.494664352

157 29.16666667 0.493773148

158 29.145 0.492881944

159 29.12333333 0.492002315

160 29.10166667 0.491111111

161 29.08 0.490231481

162 29.05833333 0.489351852

163 29.03666667 0.488472222

164 29.015 0.487592593

165 28.99333333 0.486724537

166 28.97166667 0.485856481

167 28.95 0.484988426

168 28.86612903 0.48412037

169 28.78225806 0.483263889

170 28.6983871 0.482407407

171 28.61451613 0.481550926

172 28.53064516 0.480694444

173 28.44677419 0.479849537

174 28.36290323 0.479016204

175 28.27903226 0.47818287

176 28.19516129 0.477349537

177 28.11129032 0.476527778

178 28.02741935 0.475706019

179 27.94354839 0.474884259

180 27.85967742 0.474074074

181 27.77580645 0.473275463

182 27.69193548 0.472476852

183 27.60806452 0.471689815

184 27.52419355 0.470902778

185 27.44032258 0.470127315

186 27.35645161 0.469363426

187 27.27258065 0.468599537

188 27.18870968 0.467847222

189 27.10483871 0.467106481

190 27.02096774 0.466377315

191 26.93709677 0.465648148

192 26.85322581 0.464930556

193 26.76935484 0.464224537

194 26.68548387 0.463530093

195 26.6016129 0.462847222

196 26.51774194 0.462164352

197 26.43387097 0.46150463

198 26.35 0.460856481

199 26.205 0.460219907

200 26.06 0.459583333

201 25.915 0.458969907

202 25.77 0.458368056

203 25.625 0.457777778

204 25.48 0.457210648

205 25.335 0.456643519

206 25.19 0.456099537

207 25.045 0.45556713

208 24.9 0.45505787

209 24.755 0.454548611

210 24.61 0.454074074

211 24.465 0.453599537

212 24.32 0.453148148

213 24.175 0.452708333

214 24.03 0.452291667

215 23.885 0.451898148

216 23.74 0.451516204

217 23.595 0.451145833

218 23.45 0.450798611

219 23.305 0.450474537

220 23.16 0.450162037

221 23.015 0.449872685

222 22.87 0.449606481

223 22.725 0.449363426

224 22.58 0.449131944

225 22.435 0.448923611

226 22.29 0.448738426

227 22.145 0.448564815

228 22 0.448414352

229 21.92258065 0.448287037

230 21.84516129 0.44818287

231 21.76774194 0.448101852

232 21.69032258 0.448043981

233 21.61290323 0.447997685

234 21.53548387 0.447974537

235 21.45806452 0.447974537

236 21.38064516 0.447997685

237 21.30322581 0.448043981

238 21.22580645 0.448113426

239 21.1483871 0.448194444

240 21.07096774 0.448298611

241 20.99354839 0.4484375

242 20.91612903 0.448576389

243 20.83870968 0.44875

244 20.76129032 0.448946759

245 20.60645161 0.448946759

246 20.52903226 0.449212963

247 20.4516129 0.449212963

248 20.37419355 0.449444444

249 20.29677419 0.449699074

250 20.21935484 0.449976852

251 20.14193548 0.450277778

252 20.06451613 0.450590278

253 19.98709677 0.450925926

254 19.90967742 0.451284722

255 19.83225806 0.451655093

256 19.75483871 0.452048611

257 19.67741935 0.452453704

258 19.6 0.452881944

259 19.65483871 0.453333333

260 19.70967742 0.453796296

261 19.76451613 0.454270833

262 19.81935484 0.454768519

263 19.87419355 0.455277778

264 19.92903226 0.455798611

265 19.98387097 0.456342593

266 20.03870968 0.456898148

267 20.09354839 0.457476852

268 20.1483871 0.458055556

269 20.20322581 0.458657407

270 20.25806452 0.459270833

271 20.31290323 0.459895833

272 20.36774194 0.460543981

273 20.42258065 0.46119213

274 20.47741935 0.461851852

275 20.53225806 0.462534722

276 20.58709677 0.463217593

277 20.64193548 0.463912037

278 20.69677419 0.464618056

279 20.7516129 0.465347222

280 20.80645161 0.466076389

281 20.86129032 0.466805556

282 20.91612903 0.46755787

283 20.97096774 0.468310185

284 21.02580645 0.469074074

285 21.08064516 0.469849537

286 21.13548387 0.470636574

287 21.19032258 0.471423611

288 21.24516129 0.472222222

289 21.3 0.473032407

290 21.43793103 0.473842593

291 21.57586207 0.474652778

292 21.7137931 0.475474537

293 21.85172414 0.47630787

294 21.98965517 0.477141204

295 22.12758621 0.477986111

296 22.26551724 0.478831019

297 22.40344828 0.479675926

298 22.54137931 0.480532407

299 22.67931034 0.481400463

300 22.81724138 0.482256944

301 22.95517241 0.483125

302 23.09310345 0.48400463

303 23.23103448 0.484872685

304 23.36896552 0.485752315

305 23.50689655 0.486631944

306 23.64482759 0.487523148

307 23.78275862 0.488414352

308 23.92068966 0.489305556

309 24.05862069 0.490196759

310 24.19655172 0.491087963

311 24.33448276 0.491990741

312 24.47241379 0.492893519

313 24.61034483 0.493796296

314 24.74827586 0.494699074

315 24.8862069 0.495601852

316 25.02413793 0.496516204

317 25.16206897 0.497418981

318 25.3 0.498333333

319 25.41935484 0.499236111

320 25.53870968 0.500150463

321 25.65806452 0.501064815

322 25.77741935 0.501967593

323 25.89677419 0.502881944

324 26.01612903 0.503796296

325 26.13548387 0.504710648

326 26.25483871 0.505625

327 26.37419355 0.506527778

328 26.49354839 0.50744213

329 26.61290323 0.508356481

330 26.73225806 0.509259259

331 26.8516129 0.510173611

332 26.97096774 0.511076389

333 27.09032258 0.511990741

334 27.20967742 0.512893519

335 27.32903226 0.513796296

336 27.4483871 0.514699074

337 27.56774194 0.515601852

338 27.68709677 0.51650463

339 27.80645161 0.517395833

340 27.92580645 0.518298611

341 28.04516129 0.519189815

342 28.16451613 0.520081019

343 28.28387097 0.520972222

344 28.40322581 0.521863426

345 28.52258065 0.522743056

346 28.64193548 0.523622685

347 28.76129032 0.524502315

348 28.88064516 0.52537037

349 29 0.526238426

350 29.04666667 0.527106481

351 29.09333333 0.527974537

352 29.14 0.528831019

353 29.18666667 0.5296875

354 29.23333333 0.530532407

355 29.28 0.531377315

356 29.32666667 0.532210648

357 29.37333333 0.533055556

358 29.42 0.533877315

359 29.46666667 0.534699074

360 29.51333333 0.535520833

361 29.56 0.536331019

362 29.60666667 0.53712963

363 29.65333333 0.537928241

364 29.7 0.538726852

365 29.74666667 0.539502315

366 29.79333333 0.540277778
