# Supplementary material for: An Individual-Based Model of Zebrafish Population Dynamics Accounting for Energy Dynamics
Source: PLoS One. 2015 May 4;10(5):e0125841. doi: 10.1371/journal.pone.0125841 (PMC4418570; doi:10.1371/journal.pone.0125841)
Supplement: S5 Text — (DOCX) [file pone.0125841.s007.docx]

Appendix S5. Sensitivity analysis

## Sensitivity analysis methods

The sensitivity analysis orders the inputs by importance, identifying the main contributors to the variation in the model outcome.

Sensitivity analysis of the DEB model was performed using the variance-based Sobol method (Sobol et al., 2007; Saltelli et al., 2010). It is a global and model independent sensitivity analysis method that is based on variance decomposition (quantifying the amount of variance that each parameter contributes to the unconditional variance of the model output). It can handle nonlinear and non-monotonic functions and models. Model output variances were estimated using Monte Carlo integrals. Two independent input sample $n \times p$ matrices (the “sample” matrix M1 and the “resample” matrix M2), where $n$ is the sample size ($n$ = 50,000) and p the number of parameters ($p$ = 31), were used to compute the Monte Carlo integrals. Every row in M1 and M2 represents a possible parameter combination for the model. The total cost of this analysis is $(p + 2) \times n$ model evaluations (where $p$ is the number of factors and $n$ is the random sample size). The initial parameters’ distributions were chosen according to the literature (Augustine et al., 2011) and authors’ opinion. The input parameter values for the sensitivity analysis were sampled from uniform distributions. The lower and upper limits of the uniform distributions were defined as ±10% around the posterior median value except for the *f* parameter which was distributed as *U* [0.72*,* 0.88]. These values of the *f* parameter correspond to the most frequently encountered values during experiments. First order (Si) and total Sobol’ sensitivity indices (STi) were estimated at 25, 50, 75, 100, 200, and 400 dpf for length prediction and at 100, 200, and 400 dpf for the reproduction prediction. To assess confidence intervals on the estimation of Sobol’ Indices, bootstrapping with resampling was used. The $n$ samples used for the model evaluations were 1000 times sampled with replacement, whereby, for each resampling the Si was calculated. In this way, distributions for the Si and STi values were obtained and the 95% confidence intervals were built (Nossent et al., 2011).

Sensitivity analysis of the IBM was performed using Morris method (Morris, 1991). This method is a specialised randomised One-At-a-Time sensitivity analysis in which only one input parameter is modified between two successive runs of the model. It is an efficient and reliable technique to identify non-influential inputs for a computationally costly mathematical model, where the cost of estimating other sensitivity analysis measures such as the variance-based measures is not affordable.

To perform Morris sensitivity analysis, each parameter uncertainty interval is first divided into p equally large intervals, generating a hyperspace identified by an n-dimensional p-level grid, where n is the number of parameters. A model simulation is performed based on a selection of parameters randomly sampled from the previously defined grid. Next, a single parameter is randomly selected and modified by a fixed-factor (called Δ), and a second simulation was performed. The value of Δ is fixed to p/(2(p-1)) as recommended by Morris (1991). Each factor is modified once, corresponding to n+1 runs. This process generates a trajectory in the parameter space. Then, the elementary effect, EE_y_(x_i_), of the input parameter, x_i_, on the output, y, is defined as follows:

${EE}_{y}\left( x_{i} \right)=\frac{y\left( x_{1}, \ldots, x_{i}+ \Delta, \ldots, x_{n} \right)- y\left( x_{1}, \ldots, x_{i}, \ldots, x_{n} \right)}{\Delta}$ Eq. (1)

This procedure is repeated r times (r being referred to as the sampling number hereafter), providing r elementary effects for each parameter. Thus, the Morris method depends on two parameters, p and r, and the cost of the method scales with r(n + 1). The sensitivity measures, μ_i_ and σ_i_, are the estimates of the mean and standard deviation of the elementary effects, respectively. The μ_i_ indicator assesses the overall influence of one parameter on an output and is called the first order effect. The standard deviation σ_i_ assesses the parameter’s higher order effects, such as interactions with other parameters or non-linear effects on the output. Hence, this method can determine which parameters have (i) negligible effects (non-influential parameters), (ii) linear and additive effects, or (iii) non-linear effects or interactions with other parameters. Campolongo et al. (2007) suggested the use of the mean of the absolute values of the elementary effects noted μi* to avoid minimising the inverse effects on the mean. Global index can be calculated to rank the parameters according to the Euclidian distance (Ciric et al., 2012), defined as :

$GI = \sqrt{\left( \mu_{i}^{*} \right)^{2}+ \left( \sigma i \right)^{2}}$ Eq. (2)

The cost of this analysis is $n \times(p + 1) \times r$ simulations. A drawback of the original Morris method is that, although randomised, the sampling strategy may not provide sufficient coverage of the variable space, especially when dealing with a large number of input variables. Campolongo et al. (2007) suggest an improved sampling strategy to improve the spread by creating a large number of trajectories ($r$); say from 500 to 1000 and sub-selecting a set of trajectories (in our analysis, 50 trajectories from 1000). Sensitivity analysis was then performed on the mean of the 30 repetitions ($n$) of a possible parameter combination as our model is stochastic. Supposing a normal distribution and a CV 30% of the model outputs, this number of repetition provide an uncertainty on the mean prediction equal to ±10%.

Sensitivity indices were estimated for several model outputs: the total number of fish, the frequency of adult/juvenile, the mean length of adults and juveniles at 1095, 1156, 1217, 1277, 1339, 1400 days from the beginning of the simulations (May, Jul. , Sept. , Nov. , Jan. , Mar. of the third year simulated).

## DEB sensitivity analysis results


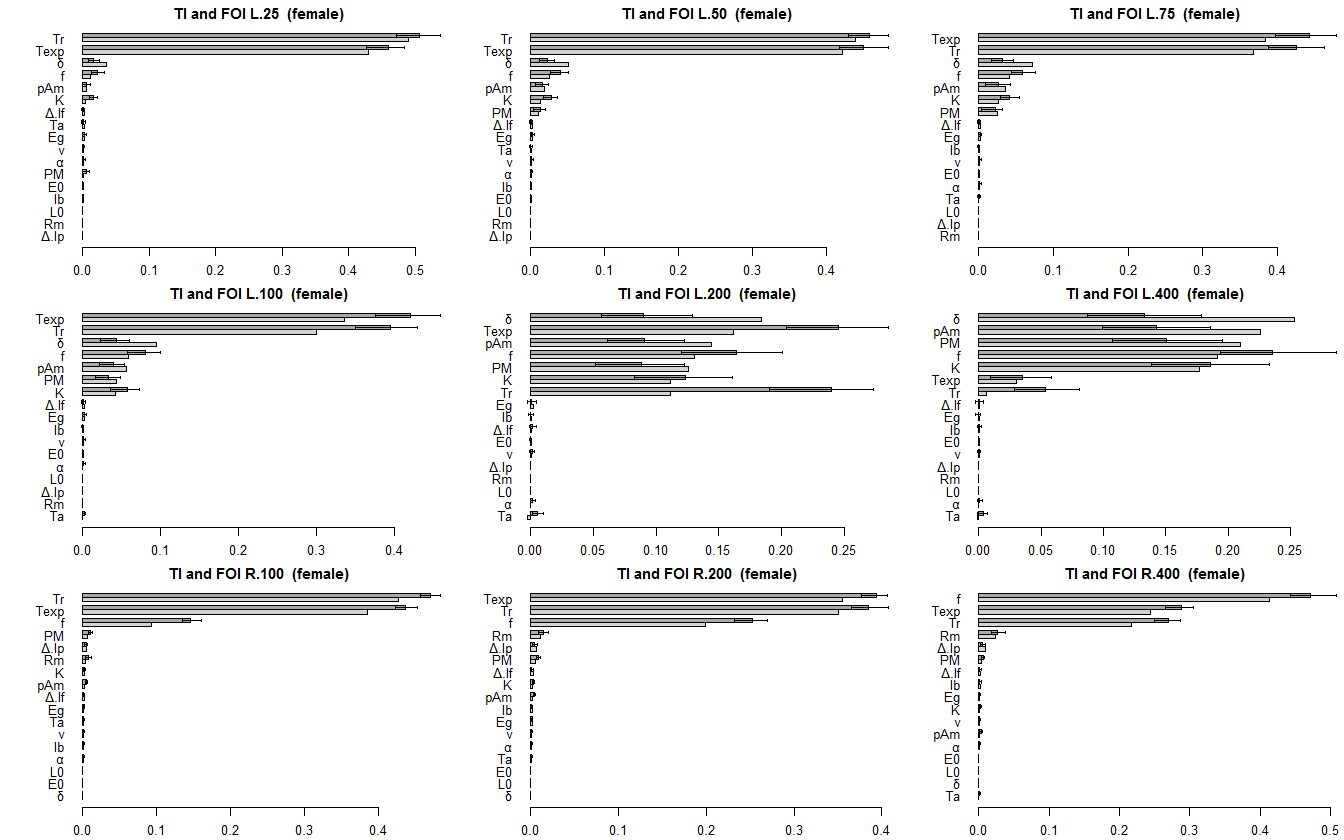


**Figure A**. Sensitivity analysis for the Zebrafish DEB model with *f* = *U* [0.72*,* 0.88]. Mean of the sensitivity indices at the different times for the growth equation (A) and reproduction equation (B). Total order indices (STi) are presented in dark grey and first order indices (Si) in light grey. Parameters were ordered according to the first order Sobol sensitivity indices

## IBM sensitivity analysis results


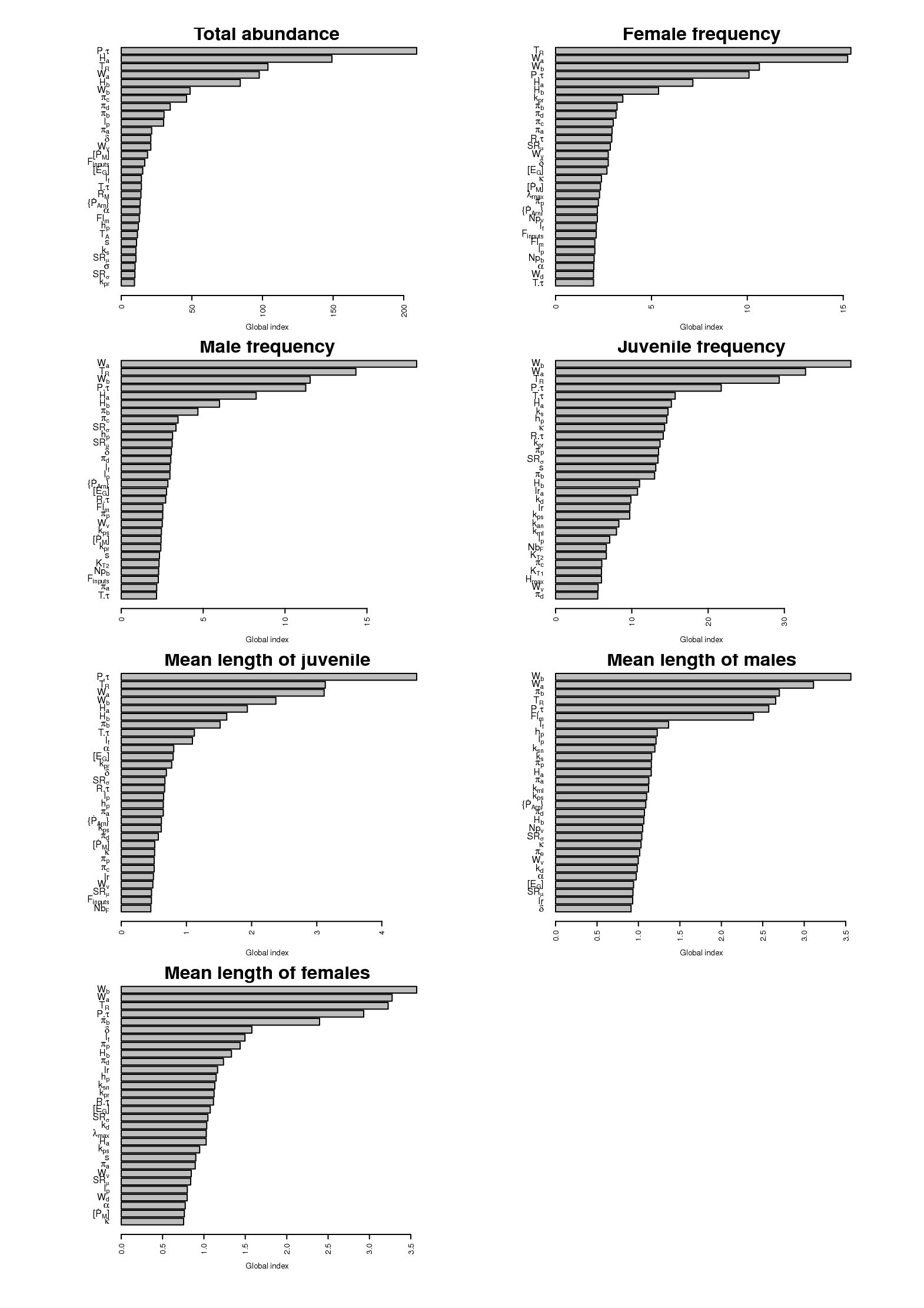


**Figure B**. Morris' global sensitivity index by outputs of the zebrafish IBM. For each model outputs, 30 most influential parameters were ordered according to the mean of the Morris' global sensitivity index measured at the different times.


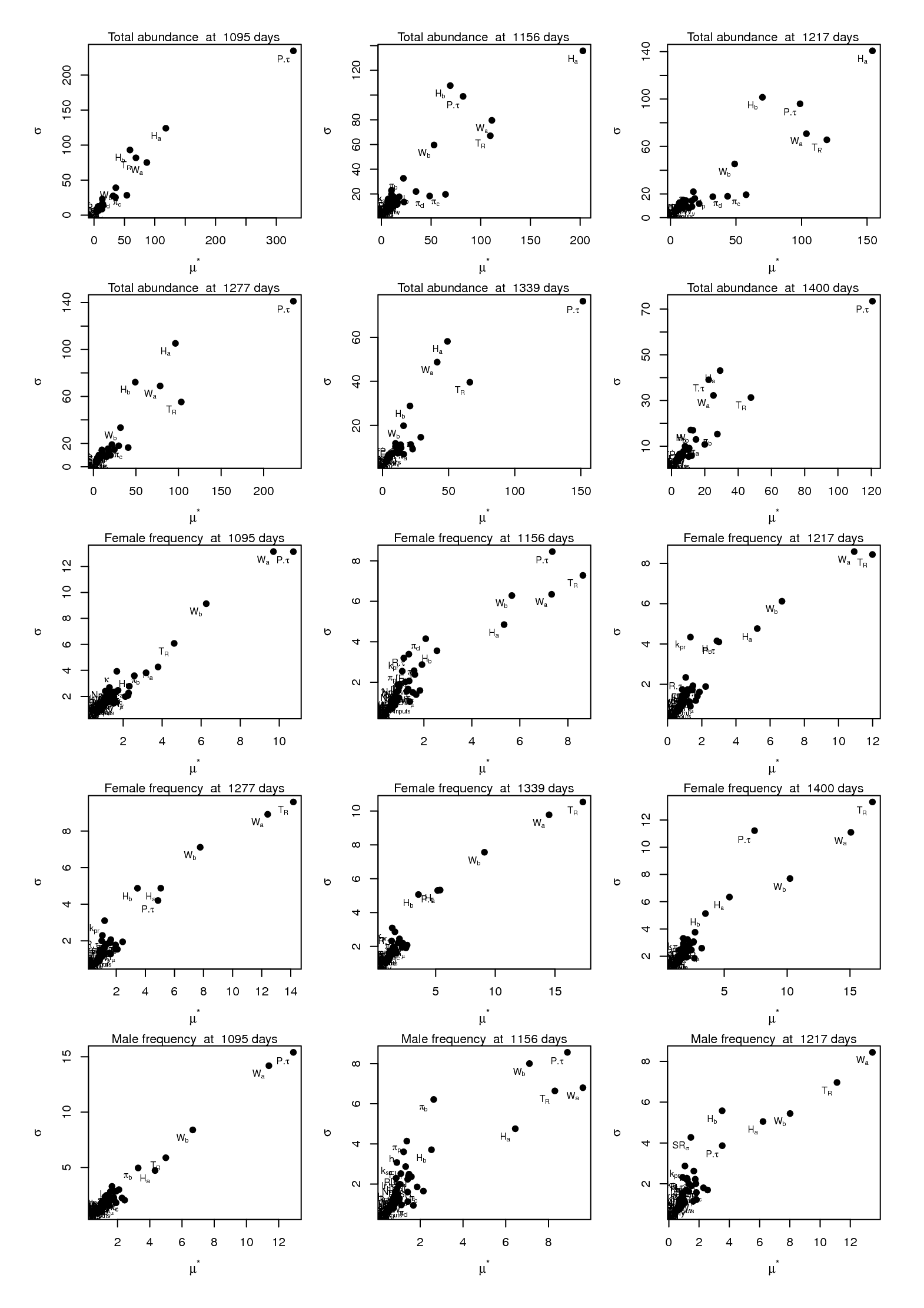


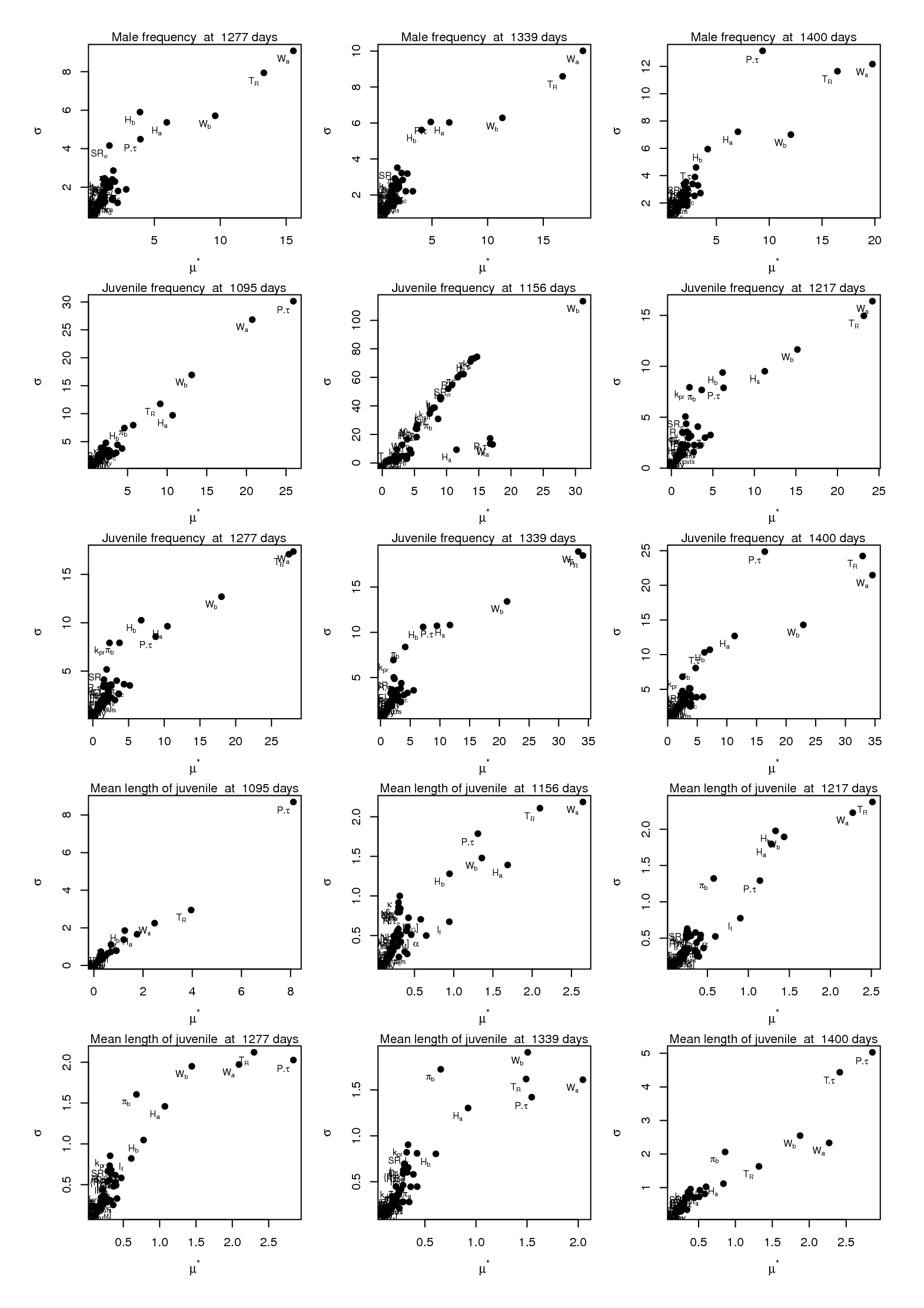


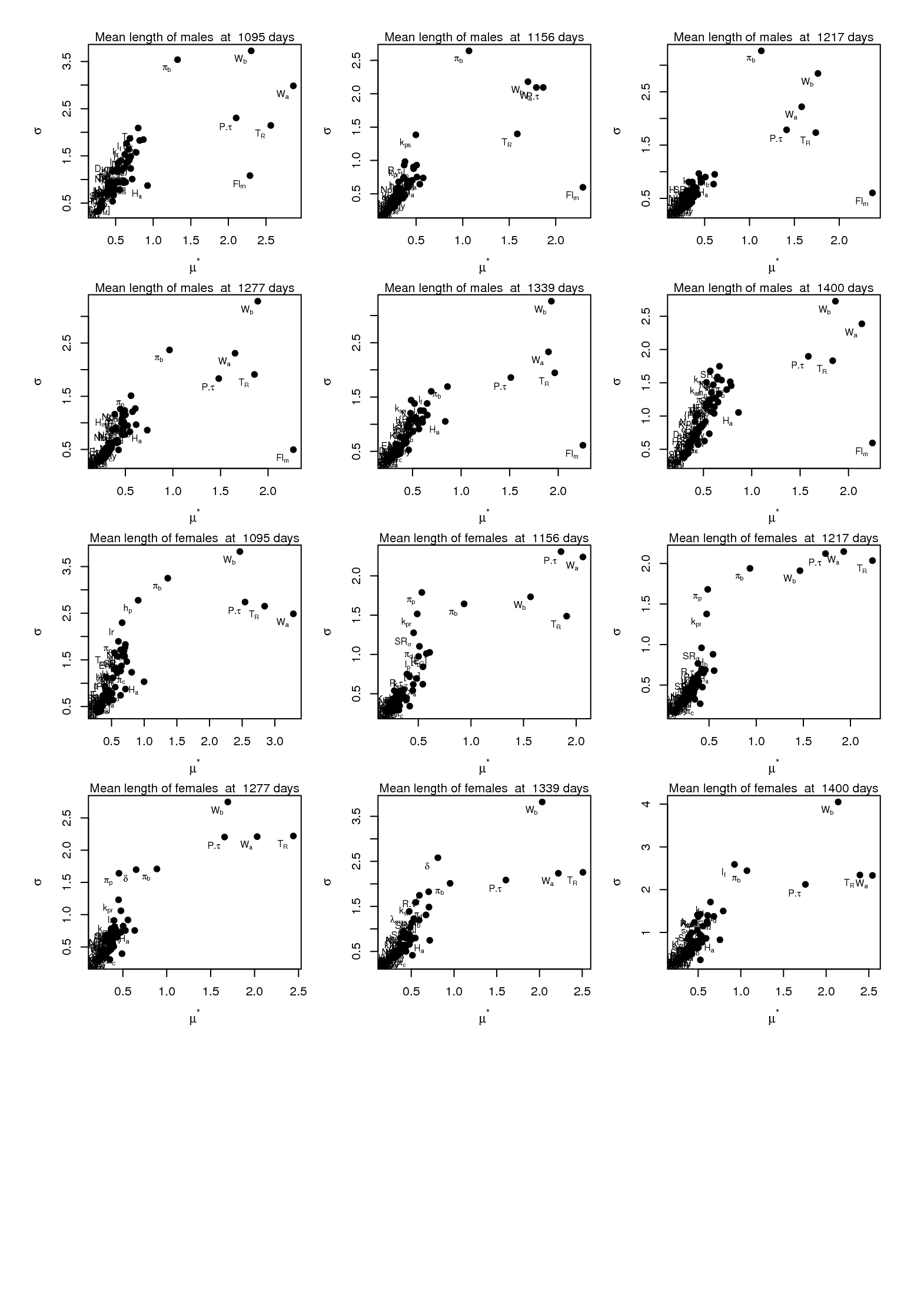


**Figure C**. Morris’ index standard deviation according to Morris’ index mean of the elementary effects calculated for the IBM of the zebrafish population dynamics. Indices were calculated for the total number of fish, the frequency of adult/juvenile, the mean length of adults and juveniles at 1095, 1156, 1217, 1277, 1339, 1400 days from the beginning of the simulations. Sensitivity analysis indices were calculated on the mean of 30 model repetitions for each parameter combination.

Augustine, S., Gagnaire, B., Floriani, M., Adam-Guillermin, C. and Kooijman, S.A.L.M., 2011. Developmental energetics of zebrafish, Danio rerio. pp. 275-283.

Campolongo, F., Cariboni, J. and Saltelli, A., 2007. An effective screening design for sensitivity. Environmental Modelling and Software, 22:1509–1518.

Ciric, C., Ciffroy, P. and Charles, S., 2012. Use of sensitivity analysis to identify influential and non-influential parameters within an aquatic ecosystem model. 246:119-130.

Morris, M.D., 1991. Factorial sampling plans for preliminary computational experiments. Technometrics, 33:161-174.

Nossent, J., Elsen, P. and Bauwens, W., 2011. Sobol' sensitivity analysis of a complex environmental model. pp. 1515-1525.

Saltelli, A., Annoni, P., Azzini, I., Campolongo, F., Ratto, M. and Tarantola, S., 2010. Variance based sensitivity analysis of model output. Design and estimator for the total sensitivity index, 259-270 pp.

Sobol, I.M., Tarantola, S., Gatelli, D., Kucherenko, S.S. and Mauntz, W., 2007. Estimating the approximation errors when fixing unessential factors in global sensitivity analysis. pp. 957-960.
